# Detailed analysis of *HTT* repeat elements in human blood using targeted amplification‐free long‐read sequencing

**DOI:** 10.1002/humu.23580

**Published:** 2018-07-12

**Authors:** Ida Höijer, Yu‐Chih Tsai, Tyson A. Clark, Paul Kotturi, Niklas Dahl, Eva‐Lena Stattin, Marie‐Louise Bondeson, Lars Feuk, Ulf Gyllensten, Adam Ameur

**Affiliations:** ^1^ Science for Life Laboratory Department of Immunology Genetics and Pathology Uppsala University Uppsala Sweden; ^2^ Pacific Biosciences Menlo Park California; ^3^ School of Public Health and Preventive Medicine Monash University Melbourne Victoria Australia

**Keywords:** amplification‐free sequencing, *HTT*, Huntington disease, No‐Amp Targeted sequencing, repeat expansion, SMRT sequencing, somatic mosaicism, targeted enrichment, targeted sequencing

## Abstract

Amplification of DNA is required as a mandatory step during library preparation in most targeted sequencing protocols. This can be a critical limitation when targeting regions that are highly repetitive or with extreme guanine–cytosine (GC) content, including repeat expansions associated with human disease. Here, we used an amplification‐free protocol for targeted enrichment utilizing the CRISPR/Cas9 system (No‐Amp Targeted sequencing) in combination with single molecule, real‐time (SMRT) sequencing for studying repeat elements in the huntingtin (*HTT*) gene, where an expanded CAG repeat is causative for Huntington disease. We also developed a robust data analysis pipeline for repeat element analysis that is independent of alignment of reads to a reference genome. The method was applied to 11 diagnostic blood samples, and for all 22 alleles the resulting CAG repeat count agreed with previous results based on fragment analysis. The amplification‐free protocol also allowed for studying somatic variability of repeat elements in our samples, without the interference of PCR stutter. In summary, with No‐Amp Targeted sequencing in combination with our analysis pipeline, we could accurately study repeat elements that are difficult to investigate using PCR‐based methods.

## 1 INTRODUCTION

Sequencing of long stretches of repeated nucleotides is notoriously difficult and yet clinically important because the length and structure of repetitive regions are diagnostic markers associated with several severe human diseases (La Spada & Taylor, 2010; Lopez Castel, Cleary, & Pearson, 2010). The optimal way to study these regions would be to sequence long molecules of native DNA without any prior amplification, because PCR dramatically reduces the chance of successfully reading through regions with extreme guanine–cytosine (GC) content or highly repetitive regions (Eid et al., 2009; Roberts, Carneiro, & Schatz, 2013; Shin et al., 2013) and may also introduce biases and chimeric molecules. In addition, sequencing of native DNA opens up for the possibility to directly study base modifications (Flusberg et al., 2010). Single molecule, real‐time (SMRT) sequencing, employed by the PacBio sequencing platforms, enables amplification‐free sequencing. Furthermore, because of the random nature of the sequencing errors, SMRT sequencing can produce highly accurate consensus sequences when given sufficient read depth (Roberts et al., 2013). Accordingly, several recent studies have demonstrated that SMRT sequencing can be used to generate high‐quality *de novo* genome assemblies of human individuals and to resolve complex repetitive regions (Seo et al., 2016; Shi et al., 2016). However, SMRT sequencing of entire human genomes at sufficient depth to resolve repeated regions is at present very expensive, and there is a need for approaches that can enrich for long DNA molecules without amplification at specific genetic loci.

Targeted enrichment, the strategy where genomic regions are selectively captured from a DNA sample before sequencing, is a cost‐effective and effort‐reducing approach in next‐generation sequencing. Most of the available methods for targeted enrichment rely on PCR during library preparation (Antson, Isaksson, Landegren, & Nilsson, 2000; Dahl et al., 2007; Gnirke et al., 2009; Tewhey et al., 2009) and are used in conjunction with short‐read sequencing technologies. While this is often a satisfactory approach, repeat elements and regions with extreme GC content (< 25% or > 65%) are major obstacles and have to be taken into consideration in the experimental design (Mertes et al., 2011). Although several targeted enrichment approaches have been adapted for long‐read SMRT sequencing, for example, using long‐range PCR (Ardui et al., 2017; Lode et al., 2017), or hybridization based approaches (Wang et al., 2015), most of these methods still include a PCR step in the sample preparation. Recently, a couple of new amplification‐free protocols have emerged, which could generate new insights into repetitive regions of the human genome predisposing to severe genetic diseases and eventually lead to novel diagnostic assays (BioRxiv: https://doi.org/10.1101/203919, BioRxiv: https://doi.org/10.1101/110163; Pham et al., 2016).

Trinucleotide repeat disorders, such as Huntington disease (HD) and Fragile X syndrome, are caused by expansion of unstable nucleotide repeats. Trinucleotide repeat expansions account for at least 22 neurological disorders, where the repeat size underlies the broad spectrum of phenotypes observed in these disorders (La Spada & Taylor, 2010; Orr & Zoghbi, 2007). HD is an autosomal dominant progressive neurodegenerative disorder caused by an expansion of a CAG repeat in the huntingtin (*HTT*) gene (MIM# 613004) on chromosome 4 (Macdonald et al., 1993). Symptoms include chorea, ataxia, and personality disorders. The onset of the disorder is usually in adulthood and a longer repeat expansion generally implies an earlier onset. The number of CAG repeats can be divided into four different size ranges that correlate with disease phenotype. Alleles up to 26 repeats are considered normal, while 27–35 repeats are intermediate alleles with potential to expand into the disease range in the next generation. Alleles with 36–39 CAG repeats are HD‐causing alleles with reduced penetrance, and the patient may or may not develop HD, and while alleles with ≥40 repeats are full‐penetrance HD‐causing alleles (Losekoot et al., 2013; Palomaki & Richards, 2012; Quarrell et al., 2012).

HD, as well as other trinucleotide repeat disorders, is typically diagnosed using PCR amplification of the repeat element, and the fragment size is determined by capillary electrophoresis. For very large expansions, Southern blotting protocols or triplet repeat primed PCR (TP‐PCR) are recommended as complementary technologies (Losekoot et al., 2013). Fragment analysis, as well as Southern blotting and TP‐PCR, is dependent upon accurate amplification and fragment sizing and does not analyze the DNA sequence itself. Studies have shown that in clinical HD analysis, 3%–13% of alleles fall outside error limits set by generally adapted best practice guidelines (Losekoot et al., 2013; Quarrell et al., 2012). An exact determination of the repeat count is important for clinical diagnostics of HD patients, in particular for the cases where repeat sizes cross borders of the repeat size ranges correlated with disease phenotypes.

Recently, we developed a protocol for targeted enrichment without amplification for use on PacBio's instruments (BioRxiv: https://doi.org/10.1101/203919). The method (named No‐Amp Targeted sequencing) employs the CRISPR/Cas9 system for directed SMRT sequencing of DNA molecules that carries the target of interest. The combination of amplification‐free target enrichment with SMRT sequencing provides a powerful tool for studying repetitive sequences and/or regions with extreme GC content. In our previous study, we described the method, how it has been optimized, and presented proof‐of‐principle data on human cell lines (BioRxiv: https://doi.org/10.1101/203919). In the present work, we apply No‐Amp Targeted sequencing on DNA isolated from blood samples from individuals subjected to clinical HD diagnostics, with the aim to study repeat elements in the *HTT* gene as well as three other clinically relevant loci: *FMR1* (MIM# 309550), *ATXN10* (MIM# 611150), and *C9orf72* (MIM# 614260). These additional loci harbor repeat expansions causative for Fragile X syndrome (CGG repeat), spinocerebellar ataxia type 10 (SCA10) (ATTCT repeat), and amyotrophic lateral sclerosis (ALS)/frontotemporal dementia (FTD) (GGGGCC repeat). In addition, we developed a robust analysis pipeline that automatically computes the repeat count on both alleles and visualizes the contents of the repeat sequence. Importantly, our analysis does not require an alignment of the sequence reads to a human reference, which is an advantage when examining these types of repeats that may often be of variable and unknown length. Previously, analysis of No‐Amp Targeting data has been alignment based and dependent on a whole panel of reference sequences, containing all possible repeat lengths, to make sure that reads with variable repeat sizes could successfully be aligned (BioRxiv: https://doi.org/10.1101/203919). Alignment‐based approaches may be suitable in many situations, but they are not ideal in cases where it is difficult to make *a priori* assumptions on the content and structure of the captured sequence. For example, this could involve regions where several different repeats of variable sizes are present, or regions containing unexpected events such as insertions. By applying our analysis tool to amplification‐free SMRT sequencing data from clinical HD samples, we obtain detailed sequence information for the *HTT* region, as well as the other captured loci, from hundreds of individual cells directly from the sequence reads. This information can be used to accurately analyze repeat elements and to study heterogeneity in repeat size within the cell population.

## 2 MATERIALS AND METHODS

### 2.1 Samples

Eleven samples that previously had undergone HD diagnostics by fragment analysis at Clinical Genetics, Uppsala University Hospital, Sweden, were included in the study. Informed consent was obtained from all individuals included in the study. All clinical investigation and genetic analyses were conducted in accordance with guidelines in the Declaration of Helsinki and the approval no. 01‐376 from the Research Ethics Committee of Uppsala University. The cohort consisted of three samples with normal alleles, three with intermediate alleles of 27–35 repeats, three with reduced penetrance 36–39 repeats, and two with alleles > 39 repeats. Genomic DNA was extracted from leukocytes according to standard procedures. For fragment analysis, five well‐characterized HD samples from Coriell CDC Repository with alleles ranging from 17 to 66 CAG repeats were included as controls (NA20207, NA20209, NA20248, NA20251, and NA20252). HEK 293 genomic DNA, used for No‐Amp Targeted sequencing, was purchased from Genscript.

### 2.2 Fragment analysis of *HTT* repeat expansions

All samples and controls were subjected to PCR with primers designed to target the CAG repeat in the *HTT* gene (5′‐CGGCGGTGGCGGCTGTTG‐3′ and 5′‐FAM‐CCTTCGAGTCCCTCAAGTCCTTC‐3′). The PCR reaction contained 30 ng genomic DNA, 1XPCR reaction buffer (GC‐rich PCR system, Sigma‐Aldrich), and 0.25 mM dNTP, 0.5 μM of primers and 0.5 U (GC‐rich enzyme; Sigma‐Aldrich). A 15‐minute enzyme activation at 96°C was followed by 40 cycles of 96°C for 15 seconds, 60°C for 30 seconds, and 72°C for 30 seconds, and a final extension at 60°C for 45 seconds. Diluted PCR products were combined with HiDi formamide and ROX500 (Thermofisher) prior to denaturation at 95°C for 5 minutes. The PCR products were separated on 3500xl Genetic Analyzer (Thermofisher), and the software GeneMarker (SoftGenetics) was used for size determination. The precision of the assay has been determined by the laboratory to ±1 repeat for alleles > 50.

### 2.3 Design of guide RNAs

**Figure 1:**
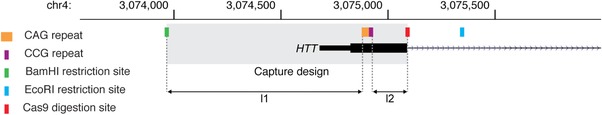
Target design for the *HTT* repeat locus. *Bam*HI is used in the fragmentation step in the library protocol, and the *Bam*HI restriction site (shown in green) determines the start of the target design. A gRNA was designed downstream of the CAG repeat (shown in orange) and CCG repeat (shown in purple) in the *HTT* gene, and the Cas9 digestion site within the gRNA design is shown in red. The complete capture design is shown within the boundaries of the gray box. The lengths between the CAG repeat and the *Bam*HI restriction site (l1) and the CCG and the Cas9 digestion site (l2) are used in downstream analysis of the repeat sizes

Guide RNAs (gRNAs) were designed using the human genome reference (GRCh38 assembly) and the CRISPR RNA configurator on Dharmacon's website (https://dharmacon.gelifesciences.com/gene-editing/crispr-rna-configurator/) with the specificity check enabled against the human genome. Guide RNAs were manually chosen with at least 200 bp flanking the repeat region and with the guide RNA's 3′ end oriented toward the repeat region. The gRNAs were also selected so that the total capture region was approximately 1 kb, where the opposite end of the region was determined by an *Eco*RI or *Bam*HI restriction site. The following guide RNAs were designed for the four targets: *C9orf72*a (5′GCAAUUCCACCAGUCGCUAG‐3′), *C9orf72*b (5′‐GCAUGAUCUCCUCGCCGGCA‐3′), *FMR1* (5′‐AGAGGCCGAACUGGGAUAAC‐3′), *HTT* (5′‐AGCGGGCCCAAACUCACGGU‐3′), and *ATXN10* (5′‐AUACAAAGGAUCAGAAUCCC‐3′). Target designs are shown in Figure 1 (*HTT*) and in Supporting Information Figure S1 (*ATXN10*, *FMR1* and *C9orf72*).

### 2.4 Library preparation and PacBio sequencing

No‐Amp Targeted sequencing libraries were prepared according to the protocol previously described by Tsai et al. (BioRxiv: https://doi.org/10.1101/203919), with minor alterations. Variable amounts of genomic DNA from all samples were subjected to digestion with 2–4 restriction enzymes (*Eco*RI, *Kpn*I, *Mfe*I, *Spe*I; New England Biolabs), at 37°C for 3 hours, in the presence of calf intestinal alkaline phosphatase (New England Biolabs) for genome complexity reduction (see Supporting InformationTable S1 for detailed information). The restriction enzymes were predicted not to cut within our target designs. Samples were fragmented with *Bam*HI‐HF and *Eco*RI‐HF (New England Biolabs) in 37°C for 3 hours followed by 20 minutes at 65°C for enzyme inactivation. Subsequently, restriction‐site–specific hairpin adapters (5′‐GATCATCTCTCTCTTTTCCTCCTCCTCCGTTGTTGTTGTTGAGAGAGAT‐3′ and 5′‐AATTATCTCTCTCTTTTCCTCCTCCTCCGTTGTTGTTGTTGAGAGAGAT‐3′) were ligated to the fragments to form SMRTbell libraries using *E. coli* DNA ligase (New England Biolabs). The adapter ligation was performed overnight at 16°C followed by 20 minutes incubation at 65°C for enzyme inactivation.

The crRNA and tracrRNA with Alt‐R modification (Intergrated DNA Technologies) were annealed in a 1:1 ratio to form gRNA that was used in the Cas9 (New England Biolabs) digestion of the SMRTbell libraries. Cas9 and gRNA in the presence of buffer were incubated at 37°C for 10 minutes, before heparin was added, and the mixture was incubated for an additional 3 minutes at 37°C. SMRTbell library was then added and incubated for 1 hour at 37°C (see Supporting Information Table S1 for sample‐gRNA combinations). EDTA was then added to terminate the reaction and the SMRTbell library was subjected to PB AMPure bead (Pacific Biosciences) purification. Hairpin adapters with a polyA‐stretch (5′‐ATCTCTCTCTTAAAAAAAAAAAAAAAAAAAAAAATTGAGAGAGAT‐3′) were ligated to the Cas9‐digested SMRTbell molecules using DNA ligase from the SMRTbell Template Prep kit 1.0 (Pacific Biosciences) to form asymmetric SMRTbell molecules containing the target of interest.

MagBeads (Pacific Biosciences) were used to enrich for asymmetric SMRTbell molecules by binding to the polyA hairpin adapter. The asymmetric SMRTbell molecules/MagBead complex was incubated under rotation at 4°C for 2 hours in MagBead Wash buffer (Pacific Biosciences), and then washed in MagBead Binding buffer (Pacific Biosciences) three times. Finally, the enriched asymmetric SMRTbells were eluted in Elution buffer (Pacific Biosciences) for 10 minutes at 65°C.

The asymmetric SMRTbell molecules were prepared for SMRT sequencing by primer annealing with standard PacBio sequencing primer lacking the polyA sequence for 1 hour at room temperature followed by AMPure PB bead (Pacific Biosciences) purification to remove excess primer. P6 polymerase was bound to the SMRTbell template/primer complex in the presence of free SMRTbell hairpin adapters to bind excess polymerase. The entire sample of enriched asymmetric SMRTbell molecules went into the primer annealing, due to unquantifiable amount of library at this point. Sequencing was performed on the PacBio RS II system using a modified MagBead One Cell Per Well protocol, C4 chemistry and 360 minutes movie time.

### 2.5 Primary analysis and alignment of PacBio reads

Asymmetric SMRTbell template sequencing data were subjected to a customized analysis pipeline for polyA‐ and conventional hairpin adapter recognition for separating subreads. The Reads of Insert tool in SMRT Portal (Pacific Biosciences) was used to create Circular Consensus Sequencing (CCS) reads from the subreads. Blasr (https://github.com/PacificBiosciences/blasr) was used to map the CCS reads to the human genome GRCh38. Mapping results were plotted in a histogram to visualize on‐target and off‐target effects.

### 2.6 Analysis of *HTT* and other repeats in PacBio data

**Figure 2:**
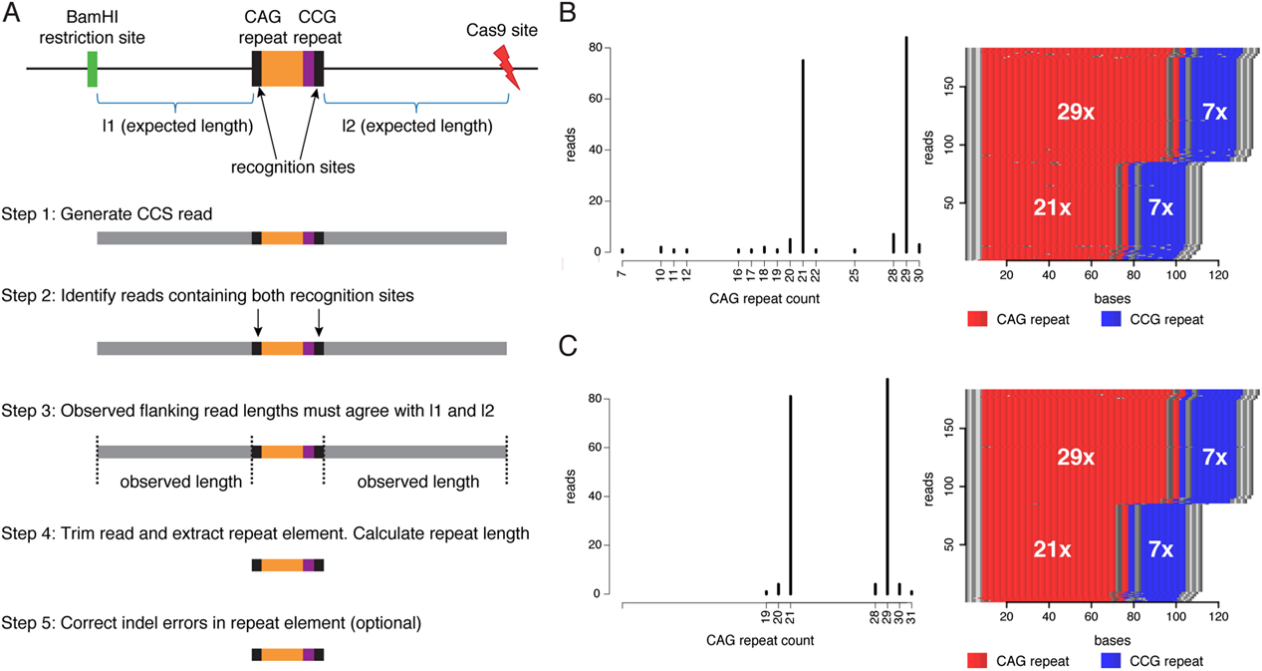
Overview of the data analysis method and visualization of results. (A) A schematic view of the *HTT* locus is shown at the top, followed by a step‐by‐step description of the analysis below. In the first step, CCS reads are generated and the figure shows a read containing the *HTT* target where the CAG repeat is represented by an orange color and the CCG repeat by a purple color, two recognition sites of length 14 bp (CCCTCAAGTCCTTC and CCTCCTCAGCTTCC) flanking the repeat are shown in black, and remaining parts of the reads upstream and downstream of the repeat are shown in gray. In step 2, reads matching the *HTT* target are identified by the recognition sites (allowing for two indel mismatches), and step 3 further requires the observed length of the upstream and downstream parts of the reads to agree with the expected lengths (l1 and l2). In step 4, the reads are trimmed so the entire repeat sequence is extracted from the read. Finally, step 5 is an optional error correction that removes indel errors within the CAG repeat sequence. (**B)** The histogram shows the distribution of CAG repeats detected in the on‐target reads for sample 10, with the two peaks at 21× and 29× representing the CAG repeat counts on the two alleles for this heterozygous individual. There is a distribution of reads having other repeat counts, and these can be explained either by somatic variation in the sample or by sequencing errors. The panel on the right shows a repeat‐content plot for the same sample. Each horizontal line corresponds to a CCS read, where CAG trinucleotides are shown in red and CCG in blue. The gray dots in the red and blue fields represent positions that contain sequences that are different from CAG and CCG. (**C)** Data from the same sample as in (B), but after indel error correction in the repeat sequences. The error correction results in a histogram with more distinct peaks at 21× and 29×, and a repeat‐content plot with fewer gray interruptions

It has previously been shown that trinucleotide repeats of at least 750 units can be accurately determined by SMRT sequencing and generation of CCS reads (Loomis et al., 2013). Because the CAG repeat in *HTT* is usually shorter than 100 units also for expanded alleles, we opted to use CCS reads instead of subreads as the basis for our analysis. To analyze the sequences in *HTT* and other repeat expansion targets, the CCS reads were used as input to a custom R script that identifies the on‐target reads, extracts the repeat element, counts the number of repeat units for each of the alleles, and visualizes the results. The program also performs an optional error correction that removes single‐base insertions or deletions within the repeats. The outline of the analysis is shown in Figure 2A. The code is available from GitHub (https://github.com/NationalGenomicsInfrastructure/HTT-repeat-analysis) along with CCS read data that can be used to execute the program.

## 3 RESULTS

### 3.1 Experimental setup

The No‐Amp Targeted sequencing approach is an amplification‐free target enrichment method that utilizes the CRISPR/Cas9 system, where the Cas9 functions as a directed endonuclease, coupled with SMRT sequencing. The method has previously been described in detail (BioRxiv: https://doi.org/10.1101/203919).

To study repeat expansions causative of HD using this amplification‐free enrichment protocol, we designed a gRNA to target the CAG and CCG repeat elements in *HTT* (Figure 1). The Cas9 digestion site was located 155 bp downstream from the CAG repeat. A *Bam*HI restriction site was located 913 bp upstream from the CAG repeat, making the complete capture design 1125 bp, under the assumption that the CAG repeat count is 19, as in the GRCh38 human reference genome. There is an SNP (rs2857935) located in the *Bam*HI site, occurring at a frequency of 34% in a cross‐section of the Swedish population (Ameur et al., 2017), which is the origin of the patients participating in this study. In samples where this particular SNP is present, the *Bam*HI restriction site closest to the CAG expansion will be unrecognizable and another *Bam*HI RE site, located 1736 bp upstream from the rs2857935, is utilized instead. No‐Amp Targeted sequencing supports multiplexing of different targets in the same assay, and to evaluate the multiplexing efficiency, we designed gRNAs to target the *C9orf72*, *FMR1*, and *ATXN10* loci (see Supporting Information Figure S1 for target designs), in addition to the original *HTT* target. Multiplexing of these gRNAs allows us to study four different repeat expansion loci in the same sample.

We first performed a number of experiments on human cell line DNA to evaluate the performance and reproducibility of the No‐Amp method, and then continued to study repeat expansions in DNA from 11 individuals who had previously been subjected to fragment analysis of the CAG repeat expansion in *HTT*. We selected samples within every repeat size range with alleles spanning from 15 to 54 CAG repeats (Table 1).

**Table 1:** Results of No‐Amp Targeted sequencing and fragment analysis in HD patient samples

	No‐Amp Targeted sequencing						Fragment analysis	
Sample	CCS reads	CCS reads on‐target	(CAG)_n_ Allele 1	(CAG)_n_ Allele 2	(CCG)_n_ Allele 1	(CCG)_n_ Allele 2	(CAG)_n_ Allele 1	(CAG)_n_ Allele 2
1	5,902	195	21	36	7	7	21	36
2	7,317	125	17	18	10	10	17	18
3	9,892	301	17	23	7	7	17	23
4	5,775	117	15	38	10	7	15	38
5	33,415	261	17	35	10	7	17	35
6	11,590	160	20	27	7	7	20	27
7	4,862	88	17	41	10	7	17	41
8	6,025	84	17	25	10	9	17	25
9	10,624	132	17	39	7	7	17	39
10	13,500	186	21	29	7	7	21	29
11	5,852	75	15	54	10	7	15	54

### 3.2 Analysis strategy

The circular topology of SMRTbell libraries enables the polymerase to repeatedly read the same molecule from both strands. When a library insert is short, up to around 5 kb, the resulting circular consensus sequences (CCS reads) are highly accurate with minimal sequencing related bias (Hestand, Van Houdt, Cristofoli, & Vermeesch, 2016; Travers, Chin, Rank, Eid, & Turner, 2010). Because our No‐Amp Targeted sequencing target designs are between 1 and 1.5 kb in length, we generated CCS reads for all samples and used CCS data in all downstream analysis.

Although several computational tools can be used to study repeat elements in next‐generation sequencing data (Dolzhenko et al., 2017; Liu, Zhang, Wang, Gu, & Wang, 2017; Tang et al., 2017), none of these has been specifically designed for the No‐Amp Targeted sequencing protocol. We therefore decided to implement our own strategy, which is outlined in Figure 2A. Our aim was to create an automated analysis method that would first identify all the on‐target reads, then extract and count the repeated units, and finally visualize the results. Importantly, we wanted all analysis steps to be performed without any alignment of reads to a reference sequence. To extract on‐target reads, we searched for specific sequences of length 14 bp flanking the start and end of the repeat unit within all CCS reads produced for a specific sample. Only reads containing both the repeat start and end elements were kept. Moreover, the lengths of the sequences upstream and downstream of the repeat element were allowed to differ at most 10% from the expected lengths from the target design, which are indicated by l1 and l2 in Figure 1. By these criteria we could very specifically extract all reads containing an expected repeat target without aligning the reads to a reference. In a subsequent step, the repeat sequences were extracted from the on‐target reads and the repeat units were counted. The results were then visualized both as a histogram and as a colored image showing the repeat structure in each on‐target read. Example results for the *HTT* repeat analysis is shown in Figure 2B.

As seen in Figure 2B, some errors were present in the data, mainly introduced by single‐base insertions/deletions in the CCS reads. We therefore developed a method that allows us to correct for indel errors within the repeat units, which is the most common type of error in SMRT sequencing data (Eid et al., 2009). The principle behind the error correction is that a repeat unit containing one indel, which is flanked at both sides with at least two correct repeat units, can be corrected. For example, when studying the CAG repeat in *HTT*, the sequence CAGCAG**CG**CAGCAG would be corrected to CACCAG**CAG**CAGCAG. Our results suggest that this error correction removes most of the indel errors and generates more accurate estimates of the repeat counts (see Figure 2C). However, the error correction should be seen an optional step as it not always advisable to modify the original reads.

### 3.3 Performance of No‐Amp Targeted sequencing

**Figure 3:**
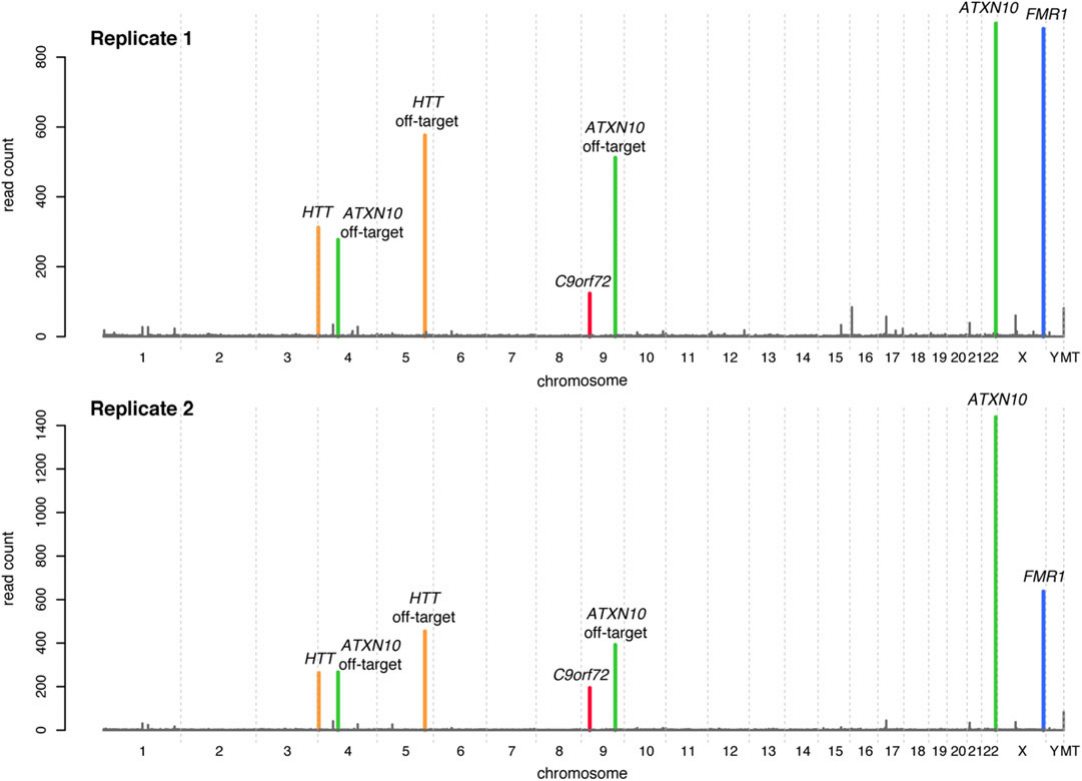
Genome‐wide coverage plots. Genome‐wide coverage plots for replicates 1 and 2, prepared from HEK 293 cell line DNA, are shown. The y‐axis shows the number of reads and the x‐axis spans over all the chromosomes in the human genome. The color of the peaks shows which gRNA the peak correlates with, green for *ATXN10*, blue for *FMR1*, orange for *HTT*, and red for *C9orf72*. In addition to the on‐target peaks for each of the gRNA, off‐target peaks are observed for the *HTT* and the *ATXN10* gRNAs

To evaluate the performance and reproducibility of the No‐Amp Targeted sequencing method, we used DNA from the commercially available HEK 293 cell line (Genscript) for CRISPR/Cas9‐targeted enrichment and sequencing. Six replicates were included in the experiment, divided over three different sequencing runs. Figure 3 shows genome‐wide coverage plots for two representative replicates. The average combined on‐target reads for the four targeted regions was 4.6%, and the average number of CCS reads per sample was 29,716 (see Supporting Information Table S2 for detailed information). The average number of on‐target reads was 209 for *HTT*, 368 for *FMR1*, 755 for *ATXN10*, and 76 for *C9orf72*.

The coverage plots in Figure 3 show peaks representing sites that were not intended to be targeted by our assay. These off‐target sites appear to be consistent over the entire set of replicates (Supporting Information Figure S2). Off‐target effects are a known consequence of the CRISPR/Cas9 system caused by locations in the genome with sufficient similarity to the gRNA target sequence to induce Cas9 activity (Fu et al., 2013). The most striking off‐target effect was found on chromosome 5, and further investigation of this site showed high homology between the *HTT* gRNA and an intronic region of the *GALNT10* gene. The sequence at this site shows a 3‐bp mismatch to the *HTT* gRNA (Supporting Information Figure S3A). Additional off‐target sites were detected on chromosomes 4 and 9. These off‐target effects were caused by homology to the *ATXN10* gRNA (Supporting Information Figure S3B and S3C).

### 3.4 Enrichment results for clinical HD samples

We further applied No‐Amp Targeted sequencing to 11 clinical HD samples, resulting in an enrichment profile over the entire genome, similar to what was obtained for the HEK 293 replicates (Supporting Information Figure S4). The average fraction of reads on target for the HD samples was 4.9%, and the average number of CCS reads per sample was 10,432. We obtained 157 on‐target reads for *HTT*, 62 for *FMR1*, 181 for *ATXN10*, and 59 for *C9orf72* on average for the 11 samples (see Supporting Information Table S3). The deviation in CCS reads between the HD samples and the HEK 293 replicates can be explained by lower amount of DNA going into the library preparation when using DNA from human blood samples (see Materials and Methods). Also, due to the limited amount of available DNA, only two REs were used for genome complexity reduction in the clinical HD samples, while four REs were used for the HEK 293 replicates. This could be another explanation for the variability in enrichment results as compared to that obtained for the HEK 293 DNA.

The off‐target effect on chromosome 5 was found in all 11 HD samples. Interestingly, only four of the samples (samples 3, 6, 7, and 9) had the off‐target effect on chromosome 9 that was observed in the HEK 293 replicates. A SNP (rs7861875) in the HEK 293 DNA increases the homology with the *ATXN10* gRNA. This SNP results in a 2‐bp mismatch in the HEK 293 samples, compared to a 3‐bp mismatch in the wild‐type allele, and this appears to be sufficient for induced Cas9 activity (Supporting Information Figure S3B). The off‐target effect found on chromosome 4 in HEK 293 was not observed in any of the clinical samples, and there is no known SNP variation that explains this variability in off‐target effect. However, it is likely that the HEK 293 cell line carries a mutation in this region that increases the homology to the gRNA design.

### 3.5 Variation in *HTT* CAG and CCG repeat size in clinical HD samples

**Figure 4:**
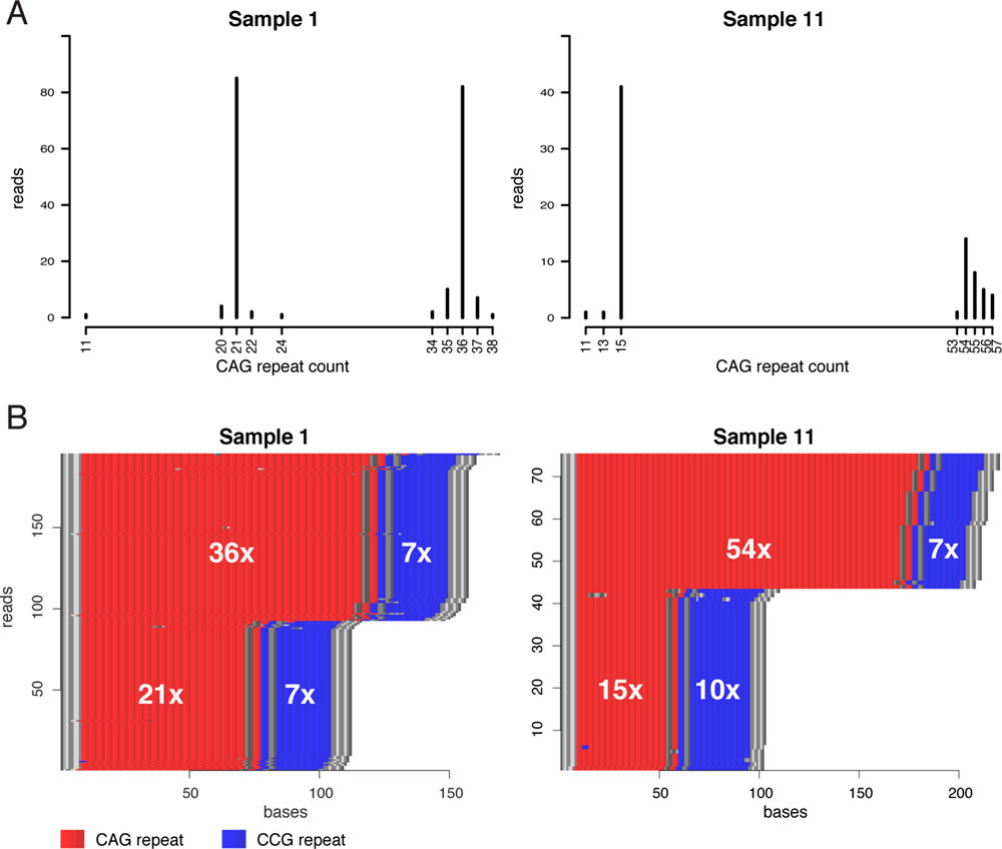
Allelic distribution of CAG repeats and repeat‐content plots for two patient samples. (A) Histograms showing the allelic distribution of CAG repeats in two patient samples. (**B)** Repeat‐content plots for the same two samples as in (A). CAG repeats are shown in red and CCG repeats are shown in blue. Sequences that are neither CAG nor CCG are shown in gray

The most prevalent CAG repeat size for every allele in the HD samples, according to our analysis, agreed with previous data from fragment analysis (Table 1). Figure 4A shows the CAG repeat distribution for two HD samples, and corresponding results for the remaining samples are shown in Supporting Information Figure S5. Interestingly, alleles with fewer repeats (e.g., sample 1) showed less repeat size variation. Conversely, alleles with large repeat sizes had a wider distribution of CAG repeats. One example of this is sample 11 that has an expanded allele ranging from 53 to 57 CAG repeats, with the highest peak at 54 (Figure 4A). A similar distribution can also be seen in fragment analysis data for sample 11, but with a very weak signal for the expanded allele (Supporting Information Figure S6). This variability indicates a somatic mosaicism of *HTT* repeat sizes, which is a known molecular event both within and in between tissues in HD patients (De Rooij, De Koning Gans, Roos, Van Ommen, & Den Dunnen, 1995; Telenius et al., 1994) and is known to be more pronounced for larger repeat sizes (Telenius et al., 1994; Veitch et al., 2007).

In addition to resolving CAG repeat sizes, we analyzed the polymorphic CCG repeat that flanks the CAG repeat. In our 11 samples, the most common CCG allele both among non‐HD‐causing and HD‐causing alleles is 7 CCG repeats, and the next most common is 10 CCG repeats, but although being polymorphic, no correlation between CCG repeat size and onset of HD has been found (Andrew, Goldberg, Theilmann, Zeisler, & Hayden, 1994). Among our sample set we found three different alleles, containing 7, 9, and 10 CCG repeats, respectively (Table 1; Supporting Information Figure S5). Fifty‐six percent (6/11) of the individuals were homozygous for either 7 or 10 repeats, and 36% (4/11) were heterozygous with 7 and 10 repeats. One individual was heterozygous with 9 and 10 repeats. The most common allele in our data is 7 CCG repeats (63%), the second most frequent is 10 CCG repeats (32%), and the 9 CCG repeat allele was the least common (5%). This distribution is in good agreement with previous studies (Agostinho Lde et al., 2012; Andrew et al., 1994). Figure 4B shows two examples of different combinations of normal and extended CAG alleles and homozygous and heterozygous CCG alleles. No apparent correlation between pathogenic CAG expansions and CCG repeat count or heterozygosity was detected. However, for all heterozygous individuals, the longer CCG repeat was flanking the shorter CAG repeat.

### 3.6 Analysis of *ATXN10*, *FMR1*, and *C9orf72* repeats

**Figure 5:**
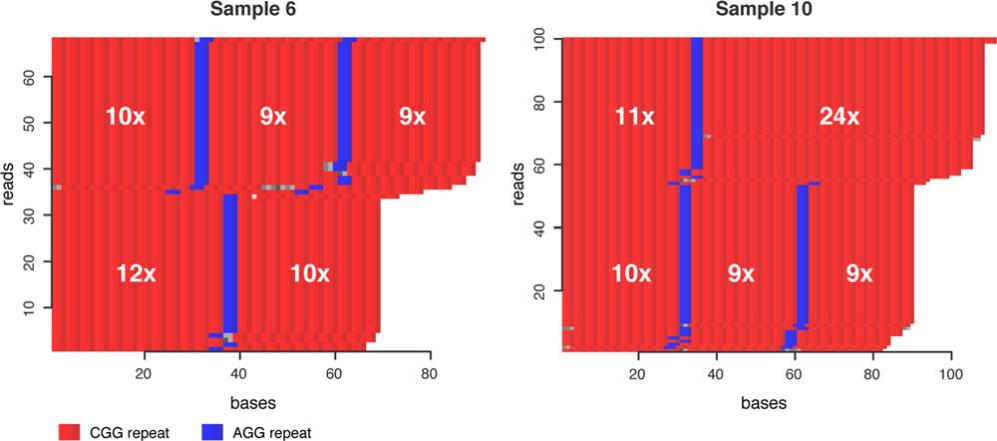
Detection of interruptions in the *FMR1* CGG repeat. Repeat‐content plots for the *FMR1* repeat sequence in two of the individuals. Sample 6 (to the left) is heterozygous, with 28 and 22 CGG repeats on the two alleles. The allele with 28 CGG repeats is interrupted by two AGG repeats (shown in blue), whereas the allele with 22 CGG repeats only contains one single AGG interruption. Sample 10 (to the right) is also heterozygous, with 35 and 28 CGG repeats on the different alleles. For this sample, the longer allele (35 × CGG) contains one AGG interruption, whereas the shorter allele (28 × CGG) contains two AGG interruptions

Even though our samples were selected for screening of the *HTT* repeat region, the multiplexing in our experiment also allowed us to analyze the captured sequences in *ATXN10*, *FMR1*, and *C9orf72* (see Supporting Information Table S3). As expected, no unusual repeat expansions were found. However, for individual 4, one of the alleles in *C9orf72* contains 15 GGGGCC repeats. This is still within the range what is generally considered normal (< 25 GGGGCC) (Cruts, Engelborghs, van der Zee, & Van Broeckhoven, 1993), but is a considerably larger repeat compared to the other GGGGCC repeats in our data set (< 8 GGGGCC). In addition to counting the number of repeats on each allele, our analysis method makes it easy to determine the presence and exact location of repeat interruptions within the *FMR1* molecules (see Figure 5). Information about repeat interruptions may in some cases have a direct clinical diagnostic value.

## 4 DISCUSSION

We have evaluated an amplification‐free targeted enrichment method for studying repeat expansions in clinically relevant samples. With the No‐Amp Targeted sequencing approach, we can obtain sequence information about the CAG and CCG repeats in the *HTT* gene without the concern of introducing bias by PCR. The complete CAG repeat region including the CCG repeat can accurately be sequenced in a single read. Although being valuable for studying repeat elements in *HTT*, the No‐Amp method may be even more powerful while analyzing larger repeat expansions, such as in *FMR1*, and other large complex genomic regions where fragment analysis and short‐read sequencing technologies struggle.

For most of the samples, over 100 reads were obtained for the *HTT* target, and we could confidently determine the size of *HTT* repeats for both alleles in all samples. Even though the number of reads on target was sufficient for analyzing the repeats in this experimental setup, there was a high background of reads derived from off‐target Cas9 activity or by unspecific pulldown of SMRTbell molecules not cleaved by Cas9. Computational gRNA design tools that allows for selection of gRNAs with minimal off‐targets sites (Heigwer, Kerr, & Boutros, 2014; Naito, Hino, Bono, & Ui‐Tei, 2015; Perez et al., 2017) could result in a more sensitive assay. Also, it would be interesting to evaluate high‐fidelity Cas9 enzymes that have proven to decrease off‐target effects while retaining on‐target activity (Kleinstiver et al., 2016). A more specific and sensitive enrichment assay would lead to reduced requirements on DNA input amounts. At present, No‐Amp Targeted sequencing requires at least 5 μg of input DNA, and this limits the use of the method to specific sample types, such as blood, where it is easy to obtain large amounts of DNA.

Variability in the number of reads on‐target was observed both between the HEK 293 replicates and in the patient samples (see Supporting Information Tables S2 and S3). The number of reads on‐target was generally lower for the blood samples that for the HEK 293 replicates. This can partly be explained by the fact that the blood samples were treated with fewer restriction enzymes for complexity reduction, as well as by differences in DNA input amount (see Materials and Methods). However, variability in the number of on‐target reads was also observed in cases where RE treatment was the same and when there were no large differences in DNA input. At present, we can only speculate about the reasons for this variation, but we believe that it is likely due to a combination of factors including sample quality and complexity, enzyme and gRNA stability, and sequencing related variabilities.

We have shown that multiplexing of targets is possible using the No‐Amp method, and we are confident that the degree of multiplexing could be even higher. Theoretically, it is possible to target nearly any region of the genome. Here, we have focused on repeat expansion disorders in humans, but this method should be applicable to a number of different types of targets in many different species. However, different sets of restriction enzymes may be needed for genome complexity reduction and target design. It is important to consider genetic variation in the experimental design because SNPs could alter restriction sites and gRNA homology and thereby affect enrichment of the target. An alternative to restriction enzyme fragmentation is random shearing, which would make the experimental design less complex, but this has yet to be fully investigated. No‐Amp Targeted sequencing also allows for multiplexing of several samples in the same run, by adding sample‐specific barcodes to the SMRTbell adapters in the first adapter ligation step of the protocol. Multiplexing of samples would decrease the input material required per sample and reduce the experimental costs. This would also require sequencing on a higher throughput instrument, such as PacBio's Sequel system, instead of the RSII system used in this study.

Previous methods for analysis of repeat expansions from amplification‐free long‐read data require the construction a panel of reference sequences containing all possible combinations of CAG and CCG repeat sizes (BioRxiv: https://doi.org/10.1101/203919). Here, we instead decided to implement a novel algorithm. Importantly, our algorithm extracts the complete repeat sequence for each individual read, thereby enabling the detection of unexpected sequences such as repeat interruptions, which are known to be of clinical importance, for example, when studying the *FMR1* repeat in Fragile X syndrome (Ardui et al., 2017). Our results reveal a mosaic pattern of repeat sizes for larger repeat expansions in *HTT*, and because this observation is based on analysis of unamplified DNA molecules, this is likely to reflect somatic variation of repeat sizes in the original DNA samples (Telenius et al., 1994; Veitch et al., 2007). The only alternative explanation would be that the additional CAG triplets are being introduced during the PacBio sequencing or during sequence analysis. Both of these explanations are highly unlikely, especially because each molecule is independently sequenced several times to create a consensus (CCS) read from several independent subreads of the molecule. In order for errors to propagate into the CCS results, the exact same erroneous CAG triplets would have to be observed in a majority of the independently sequenced subreads. We use CCS reads as the source of input to our algorithm because the CCS approach is capable of generating unbiased and highly accurate sequencing reads for repeats in our size range (Loomis et al., 2013). However, there are also some drawbacks with using CCS. Most importantly, it is difficult to study large repeat expansion molecules that are too long to generate several subreads which can be combined into a single CCS read. Thus, the CCS approach should not be attempted when expanded alleles are suspected to be of 10 kb length or more because there will be a risk of allelic dropout. For these cases, it might be necessary to use alternative methods, such as the tool recently proposed by Liu et al. (2017), which has the advantage that it can work on PacBio subreads.

Fragment analysis is a routine diagnostic genetic test for HD. Failure of amplification of large expanded alleles can lead to allelic dropout and misinterpretation of the genotype as homozygous for a normal allele (Losekoot et al., 2013; Palomaki & Richards, 2012; Potter, Spector, & Prior, 2004). Polymorphisms in primer sites could be another reason for misinterpretation of diagnostic tests using fragment analysis (Holzmann, Saecker, Epplen, & Riess, 1997; Losekoot et al., 2013; Potter et al., 2004). Heterozygosity in the flanking CCG repeat may also contribute to incorrect calling of CAG repeat sizes (Losekoot et al., 2013) if the amplicons used for sizing the CAG repeats include the CCG repeat. Southern blotting or TP‐PCR is usually used as a complement in cases where fragment analysis indicates that the individual is homozygous (Losekoot et al., 2013; Potter et al., 2004). Although our targeted enrichment method avoids biases related to PCR amplification of these complex repeats, it is still vulnerable to polymorphisms in the guide RNA sequence or restriction sites. This study represents the first time No‐Amp Targeted sequencing is used for *HTT* diagnostics, but it not yet ready to be implemented in clinical routine. For our method to replace fragment analysis, there is a need to reduce the current variation in on‐target read number, simplify the laboratory protocol, reduce the requirements on amount of input DNA, and to lower the cost. With these improvements, our method could become a powerful tool for understanding the nucleotide repeat disorders in a clinical routine setting.

Somatic variation of *HTT* repeat expansions is a known phenomenon and has been studied previously, with the largest variability observed in the regions of the brain that have most neuropathological involvement in HD (Aronin et al., 1995). As we have also seen in our results, larger repeat sizes show greater somatic variability, which is consistent with previous reports that larger repeat sizes have an earlier onset of mutation instability (Kennedy et al., 2003). Correlation between the magnitude of repeat expansion size and age of disease onset has been observed, where the most prominent somatic size mosaicism has been seen in juvenile onset of HD (Kahlem & Djian, 2000; Swami et al., 2009). The common analysis method for exploring somatic variability of CAG repeats is small‐pool PCR (SP‐PCR) (Gomes‐Pereira, Bidichandani, & Monckton, 2004) or single molecule PCR (Veitch et al., 2007), which depends on single molecule nested PCRs and detection by Southern blotting (SP‐PCR) or fragment analysis (single molecule PCR). These methods are extremely labor‐intensive, because numerous parallel PCR reactions have to be performed for each sample. Interpretation of results is affected by PCR stutter, and the true size variability may be hard to determine (Lee et al., 2010; Veitch et al., 2007). Because No‐Amp Targeted sequencing does not rely on amplification, we believe that our results provide a more accurate representation of the somatic variation compared to methods relying on bulk‐PCR. We also believe that the No‐Amp method simplifies experimental and analytical procedures in studies on somatic mosaicism of instable repeat expansions. No‐Amp Targeted sequencing also has the potential to contribute to other aspects of repeat expansion studies. A unique advantage of SMRT sequencing is the ability to directly study base modifications, such as DNA methylation, which have been shown to influence the phenotype of Fragile X (Usdin et al., 2014) and might also be relevant in other repeat expansion disorders.

In conclusion, we have successfully applied a novel amplification‐free targeted enrichment method to study the trinucleotide repeat in *HTT* in clinical HD samples, as well as the three additional loci *ATXN10*, *FMR1*, and *C9orf72*. Our mapping‐independent software allowed us to confidently analyze the unstable *HTT* repeat and to study repeat sequence variations such as interruptions in the *FMR1* repeat. The PCR‐free methodology makes it possible to study somatic and allelic variation without any influence of PCR stutter or other amplification‐related biases.

## 5 AVAILABILITY

The data analysis code, CCS data, and user instructions are available from the following URL: https://github.com/NationalGenomicsInfrastructure/HTT-repeat-analysis. For the 11 patient samples, only CCS reads corresponding to the four target sites (*HTT*, *FMR1*, *ATXN10*, and *C9orf72*) are available from GitHub.

## Supporting information


**Figure S1**. Target designs. No‐Amp Targeted sequencing target designs for the ATXN10, FMR1 and C9orf72 repeat regions.
**Figure S2**. Coverage plots for replicates. Genome‐wide coverage plots for the HEK 293 replicates.
**Figure S3**. Off‐target effects. Off‐target effects caused by gRNA homology to other loci in the genome.
**Figure S4**. Coverage plots for HD samples. Genome‐wide coverage plots for the 11 HD samples.
**Figure S5**. Allelic distribution of CAG repeats and repeat‐content plots for 11 HD samples.
**Figure S6**. Fragment analysis results for sample 11.
**Table S1**. Sample library preparation information.
**Table S2**. Reads on‐target and total CCS reads for HEK 293 replicates.
**Table S3**. Reads on‐target and total CCS reads for 11 patient samples.

## References

[humu23580-bib-0001] Agostinho Lde, A. , Rocha, C. F. , Medina‐Acosta, E. , Barboza, H. N. , da Silva, A. F. , Pereira, S. P. , … Paiva, C. L. (2012). Haplotype analysis of the CAG and CCG repeats in 21 Brazilian families with Huntington's disease. Journal of Human Genetics, 57(12)796–803. 10.1038/jhg.2012.120 23051704

[humu23580-bib-0002] Ameur, A. , Dahlberg, J. , Olason, P. , Vezzi, F. , Karlsson, R. , Martin, M. , … Gyllensten, U. (2017). SweGen: A whole‐genome data resource of genetic variability in a cross‐section of the Swedish population. European Journal of Human Genetics, 25(11), 1253–1260. 10.1038/ejhg.2017.130 28832569 PMC5765326

[humu23580-bib-0003] Andrew, S. E. , Goldberg, Y. P. , Theilmann, J. , Zeisler, J. , & Hayden, M. R. (1994). A CCG repeat polymorphism adjacent to the CAG repeat in the Huntington disease gene: Implications for diagnostic accuracy and predictive testing. Human Molecular Genetics, 3(1), 65–67. 8162053 10.1093/hmg/3.1.65

[humu23580-bib-0004] Antson, D. O. , Isaksson, A. , Landegren, U. , & Nilsson, M. (2000). PCR‐generated padlock probes detect single nucleotide variation in genomic DNA. Nucleic Acids Res., 28(12), E58. 10871381 10.1093/nar/28.12.e58 PMC102743

[humu23580-bib-0005] Ardui, S. , Race, V. , Zablotskaya, A. , Hestand, M. S. , Van Esch, H. , Devriendt, K. , … Vermeesch, J. R. (2017). Detecting AGG interruptions in male and female FMR1 premutation carriers by single‐molecule sequencing. Human Mutation, 38(3), 324–331. 10.1002/humu.23150 27883256

[humu23580-bib-0006] Aronin, N. , Chase, K. , Young, C. , Sapp, E. , Schwarz, C. , Matta, N. , … et al. (1995). CAG expansion affects the expression of mutant Huntingtin in the Huntington's disease brain. Neuron, 15(5), 1193–1201. 7576661 10.1016/0896-6273(95)90106-x

[humu23580-bib-0007] Cruts, M. , Engelborghs, S. , van der Zee, J. , & Van Broeckhoven, C. (1993). C9orf72‐related amyotrophic lateral sclerosis and frontotemporal dementia In Adam M. P., Ardinger H. H., Pagon R. A., Wallace S. E., Bean L. J. H., Stephens K., & Amemiya A. (Eds.), GeneReviews((R)). Seattle, WA: University of Washington, Seattle. 25577942

[humu23580-bib-0008] Dahl, F. , Stenberg, J. , Fredriksson, S. , Welch, K. , Zhang, M. , Nilsson, M. , … Ji, H. (2007). Multigene amplification and massively parallel sequencing for cancer mutation discovery. Proc Natl Acad Sci U S A, 104(22), 9387–9392. 10.1073/pnas.0702165104 17517648 PMC1871563

[humu23580-bib-0009] De Rooij, K. E. , De Koning Gans, P. A. , Roos, R. A. , Van Ommen, G. J. , & Den Dunnen, J. T. (1995). Somatic expansion of the (CAG)n repeat in Huntington disease brains. Human Genetics, 95(3), 270–274. 7868117 10.1007/BF00225192

[humu23580-bib-0010] Dolzhenko, E. , van Vugt, J. , Shaw, R. J. , Bekritsky, M. A. , van Blitterswijk, M. , Narzisi, G. , … Eberle, M. A. (2017). Detection of long repeat expansions from PCR‐free whole‐genome sequence data. Genome Research, 27(11), 1895–1903. 10.1101/gr.225672.117 28887402 PMC5668946

[humu23580-bib-0011] Eid, J. , Fehr, A. , Gray, J. , Luong, K. , Lyle, J. , Otto, G. , … Turner, S. (2009). Real‐time DNA sequencing from single polymerase molecules. Science, 323(5910), 133–138. 10.1126/science.1162986 19023044

[humu23580-bib-0012] Flusberg, B. A. , Webster, D. R. , Lee, J. H. , Travers, K. J. , Olivares, E. C. , Clark, T. A. , … Turner, S. W. (2010). Direct detection of DNA methylation during single‐molecule, real‐time sequencing. Nature Methods, 7(6), 461–465. 10.1038/nmeth.1459 20453866 PMC2879396

[humu23580-bib-0013] Fu, Y. , Foden, J. A. , Khayter, C. , Maeder, M. L. , Reyon, D. , Joung, J. K. , & Sander, J. D. (2013). High‐frequency off‐target mutagenesis induced by CRISPR‐Cas nucleases in human cells. Nature Biotechnology, 31(9), 822–826. 10.1038/nbt.2623 PMC3773023 23792628

[humu23580-bib-0014] Gnirke, A. , Melnikov, A. , Maguire, J. , Rogov, P. , LeProust, E. M. , Brockman, W. , … Nusbaum, C. (2009). Solution hybrid selection with ultra‐long oligonucleotides for massively parallel targeted sequencing. Nature Biotechnology, 27(2), 182–189. 10.1038/nbt.1523 PMC2663421 19182786

[humu23580-bib-0015] Gomes‐Pereira, M. , Bidichandani, S. I. , & Monckton, D. G. (2004). Analysis of unstable triplet repeats using small‐pool polymerase chain reaction. Methods in Molecular Biology, 277, 61–76. 10.1385/1-59259-804-8:061 15201449

[humu23580-bib-0016] Heigwer, F. , Kerr, G. , & Boutros, M. (2014). E‐CRISP: Fast CRISPR target site identification. Nature Methods, 11(2), 122–123. 10.1038/nmeth.2812 24481216

[humu23580-bib-0017] Hestand, M. S. , Van Houdt, J. , Cristofoli, F. , & Vermeesch, J. R. (2016). Polymerase specific error rates and profiles identified by single molecule sequencing. Mutation Research, 784–785, 39–45. 10.1016/j.mrfmmm.2016.01.003 26829216

[humu23580-bib-0018] Holzmann, C. , Saecker, A. M. , Epplen, J. T. , & Riess, O. (1997). Avoiding errors in the diagnosis of (CAG)n expansion in the huntingtin gene. Journal of Medical Genetics, 34(3), 264. 9132504 10.1136/jmg.34.3.264 PMC1050907

[humu23580-bib-0019] Kahlem, P. , & Djian, P. (2000). The expanded CAG repeat associated with juvenile Huntington disease shows a common origin of most or all neurons and glia in human cerebrum. Neuroscience Letters, 286(3), 203–207. 10832020 10.1016/s0304-3940(00)01029-6

[humu23580-bib-0020] Kennedy, L. , Evans, E. , Chen, C. M. , Craven, L. , Detloff, P. J. , Ennis, M. , & Shelbourne, P. F. (2003). Dramatic tissue‐specific mutation length increases are an early molecular event in Huntington disease pathogenesis. Human Molecular Genetics, 12(24), 3359–3367. 10.1093/hmg/ddg352 14570710

[humu23580-bib-0021] Kleinstiver, B. P. , Pattanayak, V. , Prew, M. S. , Tsai, S. Q. , Nguyen, N. T. , Zheng, Z. , & Joung, J. K. (2016). High‐fidelity CRISPR‐Cas9 nucleases with no detectable genome‐wide off‐target effects. Nature, 529(7587), 490–495. 10.1038/nature16526 26735016 PMC4851738

[humu23580-bib-0022] La Spada, A. R. , & Taylor, J. P. (2010). Repeat expansion disease: Progress and puzzles in disease pathogenesis. Nature Reviews Genetics, 11(4), 247–258. 10.1038/nrg2748 PMC4704680 20177426

[humu23580-bib-0023] Lee, J. M. , Zhang, J. , Su, A. I. , Walker, J. R. , Wiltshire, T. , Kang, K. , … Wheeler, V. C. (2010). A novel approach to investigate tissue‐specific trinucleotide repeat instability. Bmc Systems Biology, 4, 29 10.1186/1752-0509-4-29 20302627 PMC2856555

[humu23580-bib-0024] Liu, Q. , Zhang, P. , Wang, D. , Gu, W. , & Wang, K. (2017). Interrogating the “unsequenceable” genomic trinucleotide repeat disorders by long‐read sequencing. Genome Med, 9(1), 65 10.1186/s13073-017-0456-7 28720120 PMC5514472

[humu23580-bib-0025] Lode, L. , Ameur, A. , Coste, T. , Menard, A. , Richebourg, S. , Gaillard, J. B. , … Soussi, T. (2017). Single‐molecule DNA sequencing of acute myeloid leukemia and myelodysplastic syndromes with multiple TP53 alterations. Haematologica, 10.3324/haematol.2017.176719 PMC5777207 29079597

[humu23580-bib-0026] Loomis, E. W. , Eid, J. S. , Peluso, P. , Yin, J. , Hickey, L. , Rank, D. , … Hagerman, P. J. (2013). Sequencing the unsequenceable: Expanded CGG‐repeat alleles of the fragile X gene. Genome Research, 23(1), 121–128. 10.1101/gr.141705.112 23064752 PMC3530672

[humu23580-bib-0027] Lopez Castel, A. , Cleary, J. D. , & Pearson, C. E. (2010). Repeat instability as the basis for human diseases and as a potential target for therapy. Nature Reviews Molecular Cell Biology, 11(3), 165–170. 10.1038/nrm2854 20177394

[humu23580-bib-0028] Losekoot, M. , van Belzen, M. J. , Seneca, S. , Bauer, P. , Stenhouse, S. A. , & Barton, D. E. & European Molecular Genetic Quality, N . (2013). EMQN/CMGS best practice guidelines for the molecular genetic testing of Huntington disease. European Journal of Human Genetics, 21(5), 480–486. 10.1038/ejhg.2012.200 22990145 PMC3641377

[humu23580-bib-0029] Macdonald, M. E. , Ambrose, C. M. , Duyao, M. P. , Myers, R. H. , Lin, C. , Srinidhi, L. , … Harper, P. S. (1993). A novel gene containing a trinucleotide repeat that is expanded and unstable on huntingtons‐disease chromosomes. Cell, 72(6), 971–983. 10.1016/0092-8674(93)90585-E 8458085

[humu23580-bib-0030] Mertes, F. , Elsharawy, A. , Sauer, S. , van Helvoort, J. M. , van der Zaag, P. J. , Franke, A. , … Brookes, A. J. (2011). Targeted enrichment of genomic DNA regions for next‐generation sequencing. Brief Funct Genomics, 10(6), 374–386. 10.1093/bfgp/elr033 22121152 PMC3245553

[humu23580-bib-0031] Naito, Y. , Hino, K. , Bono, H. , & Ui‐Tei, K. (2015). CRISPRdirect: Software for designing CRISPR/Cas guide RNA with reduced off‐target sites. Bioinformatics, 31(7), 1120–1123. 10.1093/bioinformatics/btu743 25414360 PMC4382898

[humu23580-bib-0032] Orr, H. T. , & Zoghbi, H. Y. (2007). Trinucleotide repeat disorders. Annual Review of Neuroscience, 30, 575–621. 10.1146/annurev.neuro.29.051605.113042 17417937

[humu23580-bib-0033] Palomaki, G. E. , & Richards, C. S. (2012). Assessing the analytic validity of molecular testing for Huntington disease using data from an external proficiency testing survey. Genetics in Medicine, 14(1), 69–75. 10.1038/gim.0b013e3182310bb5 22237433

[humu23580-bib-0034] Perez, A. R. , Pritykin, Y. , Vidigal, J. A. , Chhangawala, S. , Zamparo, L. , Leslie, C. S. , & Ventura, A. (2017). GuideScan software for improved single and paired CRISPR guide RNA design. Nature Biotechnology, 35(4), 347–349. 10.1038/nbt.3804 PMC5607865 28263296

[humu23580-bib-0035] Pham, T. T. , Yin, J. , Eid, J. S. , Adams, E. , Lam, R. , Turner, S. W. , … Hanes, J. W. (2016). Single‐locus enrichment without amplification for sequencing and direct detection of epigenetic modifications. Molecular Genetics and Genomics, 291(3), 1491–1504. 10.1007/s00438-016-1167-2 26825750

[humu23580-bib-0036] Potter, N. T. , Spector, E. B. , & Prior, T. W. (2004). Technical standards and guidelines for Huntington disease testing. Genetics in Medicine, 6(1), 61–65. doi: https://doi.org/10.109701.GIM.0000106165.74751.15 14726813 10.1097/01.gim.0000106165.74751.15

[humu23580-bib-0037] Quarrell, O. W. , Handley, O. , O'Donovan, K. , Dumoulin, C. , Ramos‐Arroyo, M. , Biunno, I. , … European Huntington's Disease, N. (2012). Discrepancies in reporting the CAG repeat lengths for Huntington's disease. European Journal of Human Genetics, 20(1), 20–26. 10.1038/ejhg.2011.136 21811303 PMC3234505

[humu23580-bib-0038] Roberts, R. J. , Carneiro, M. O. , & Schatz, M. C. (2013). The advantages of SMRT sequencing. Genome Biology, 14(7), 405 10.1186/gb-2013-14-6-405 23822731 PMC3953343

[humu23580-bib-0039] Seo, J. S. , Rhie, A. , Kim, J. , Lee, S. , Sohn, M. H. , Kim, C. U. , … Kim, C. (2016). De novo assembly and phasing of a Korean human genome. Nature, 538(7624), 243–247. 10.1038/nature20098 27706134

[humu23580-bib-0040] Shi, L. , Guo, Y. , Dong, C. , Huddleston, J. , Yang, H. , Han, X. , … Wang, K. (2016). Long‐read sequencing and de novo assembly of a Chinese genome. Nat Commun, 7, 12065 10.1038/ncomms12065 27356984 PMC4931320

[humu23580-bib-0041] Shin, S. C. , Ahn, D. H. , Kim, S. J. , Lee, H. , Oh, T. J. , Lee, J. E. , & Park, H. (2013). Advantages of single‐molecule real‐time sequencing in high‐GC content genomes. Plos One, 8(7), e68824 10.1371/journal.pone.0068824 23894349 PMC3720884

[humu23580-bib-0042] Swami, M. , Hendricks, A. E. , Gillis, T. , Massood, T. , Mysore, J. , Myers, R. H. , & Wheeler, V. C. (2009). Somatic expansion of the Huntington's disease CAG repeat in the brain is associated with an earlier age of disease onset. Human Molecular Genetics, 18(16), 3039–3047. 10.1093/hmg/ddp242 19465745 PMC2714728

[humu23580-bib-0043] Tang, H. , Kirkness, E. F. , Lippert, C. , Biggs, W. H. , Fabani, M. , Guzman, E. , … Telenti, A. (2017). Profiling of short‐tandem‐repeat disease alleles in 12,632 human whole genomes. American Journal of Human Genetics, 101(5), 700–715. 10.1016/j.ajhg.2017.09.013 29100084 PMC5673627

[humu23580-bib-0044] Telenius, H. , Kremer, B. , Goldberg, Y. P. , Theilmann, J. , Andrew, S. E. , Zeisler, J. … et al. (1994). Somatic and gonadal mosaicism of the Huntington disease gene CAG repeat in brain and sperm. Nature Genetics, 6(4), 409–414. 10.1038/ng0494-409 8054984

[humu23580-bib-0045] Tewhey, R. , Warner, J. B. , Nakano, M. , Libby, B. , Medkova, M. , David, P. H. , … Frazer, K. A. (2009). Microdroplet‐based PCR enrichment for large‐scale targeted sequencing. Nature Biotechnology, 27(11), 1025–1031. 10.1038/nbt.1583 PMC2779736 19881494

[humu23580-bib-0046] Travers, K. J. , Chin, C. S. , Rank, D. R. , Eid, J. S. , & Turner, S. W. (2010). A flexible and efficient template format for circular consensus sequencing and SNP detection. Nucleic Acids Res., 38(15), e159 10.1093/nar/gkq543 20571086 PMC2926623

[humu23580-bib-0047] Usdin, K. , Hayward, B. E. , Kumari, D. , Lokanga, R. A. , Sciascia, N. , & Zhao, X. N. (2014). Repeat‐mediated genetic and epigenetic changes at the FMR1 locus in the fragile X‐related disorders. Front Genet, 5, 226 10.3389/fgene.2014.00226 25101111 PMC4101883

[humu23580-bib-0048] Veitch, N. J. , Ennis, M. , McAbney, J. P. , Project, U. S.‐V. C. R. , Shelbourne, P. F. , & Monckton, D. G. (2007). Inherited CAG.CTG allele length is a major modifier of somatic mutation length variability in Huntington disease. Dna Repair, 6(6), 789–796. 10.1016/j.dnarep.2007.01.002 17293170

[humu23580-bib-0049] Wang, M. , Beck, C. R. , English, A. C. , Meng, Q. , Buhay, C. , Han, Y. , … Gibbs, R. A. (2015). PacBio‐LITS: A large‐insert targeted sequencing method for characterization of human disease‐associated chromosomal structural variations. Bmc Genomics [Electronic Resource], 16, 214 10.1186/s12864-015-1370-2 PMC4376517 25887218

