# Supplementary material for: Detailed analysis of HTT repeat elements in human blood using targeted amplification‐free long‐read sequencing
Source: Hum Mutat. 2018 Jul 12;39(9):1262–72. doi: 10.1002/humu.23580 (PMC6175010; doi:10.1002/humu.23580)
Supplement: Supplementary file 1 — Figure S1. Target designs. No‐Amp Targeted sequencing target designs for the ATXN10, FMR1 and C9orf72 repeat regions. Figure S2. Coverage plots for replicates. Genome‐wide coverage plots for the HEK 293 replicates. Figure S3. Off‐target effects. Off‐target effects caused by gRNA homology to other loci in the genome. Figure S4. Coverage plots for HD samples. Genome‐wide coverage plots for the 11 HD samples. Figure S5. Allelic distribution of CAG repeats and repeat‐content plots for 11 HD samples. Figure S6. Fragment analysis results for sample 11. Table S1. Sample library preparation information. Table S2. Reads on‐target and total CCS reads for HEK 293 replicates. Table S3. Reads on‐target and total CCS reads for 11 patient samples. [file HUMU-39-1262-s001.pdf]

Supporting Material and Methods

Detailed analysis of *HTT* repeat elements in human blood using targeted amplification-free long-read sequencing

Ida Höijer, Yu-Chih Tsai, Tyson A. Clark, Paul Kotturi, Niklas Dahl, Eva-Lena Stattin, Marie-Louise Bondeson, Lars Feuk, Ulf Gyllensten and Adam Ameur.

Supporting Figures

Figure S1. Target designs. No-amp targeted sequencing target designs for the *ATXN10*, *FMR1* and *C9orf72* repeat regions.

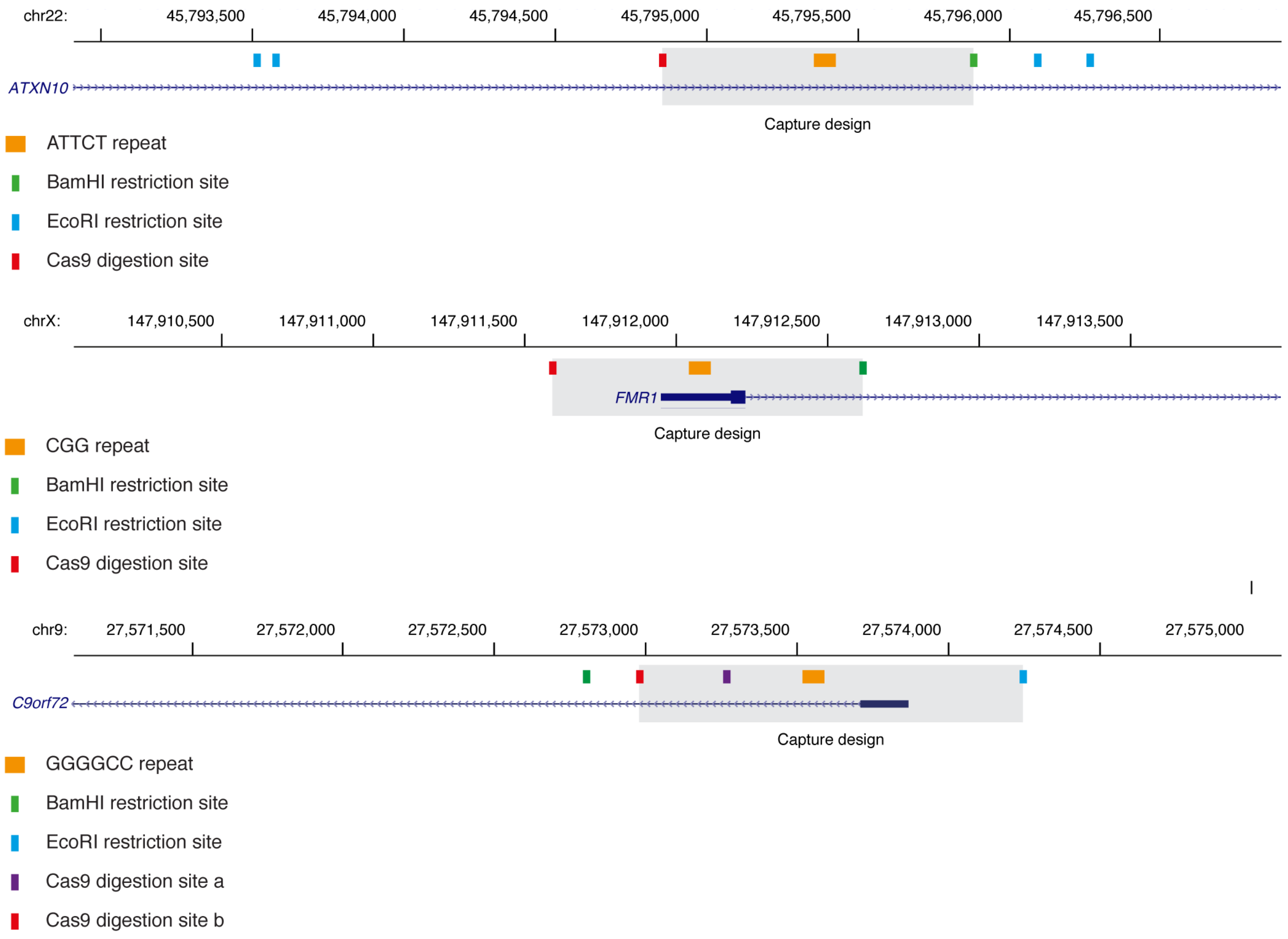

Figure S2. Coverage plots for replicates. Genome wide coverage plots for the HEK 293 replicates.

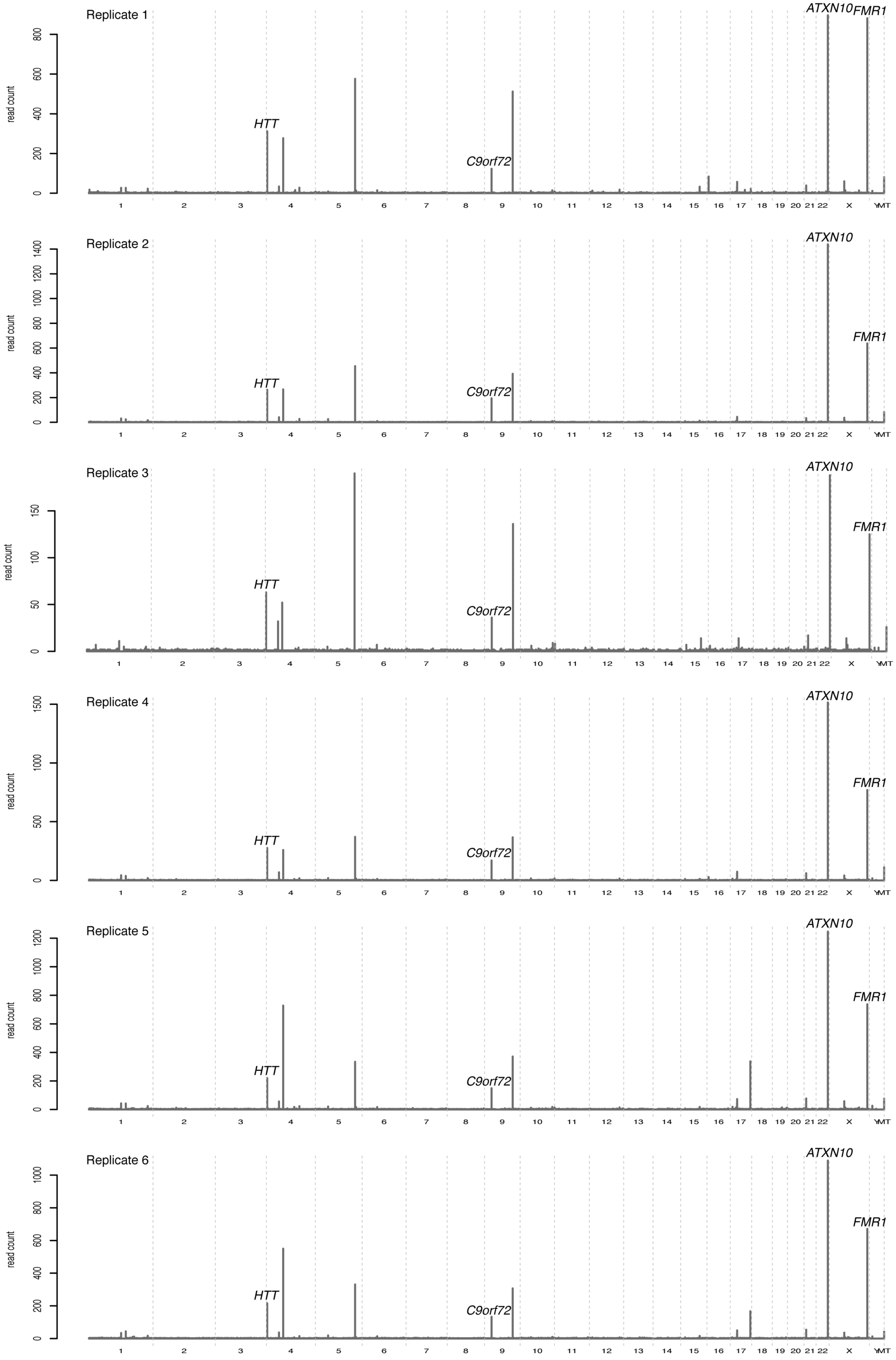

Figure S3. Off-target effects. Off-target effects caused by gRNA homology to other loci in the genome. A) Off-target effect caused by homology to the *HTT* gRNA in chromosome 5. **B)** Off-target effect in chromosome 9. Only genomes with SNP rs7861875 show the off-target effect caused by homology to the *ATXN10* gRNA**. C)** Off-target effect in in chromosome 4 caused by homology to the *ATXN10* gRNA. Genomic coordinates refer to the GRCh38 reference genome.

A

```
chr5:154,409,392-154,409,411  AGAAGGCCTAAACTCACGGT
                              ||..||||.|||||||||||
HTT gRNA                      AGCGGGCCCAAACUCACGGU
```

B

```
                                rs7861875
                                   G
chr9:109,570,949-109,570,968  ATACAACATATCAGAATCCC
                              ||||||...|||||||||||
ATXN10 gRNA                   AUACAAAGGAUCAGAAUCCC
```

C

```
chr4:65,163,126-65,163,145    TCACAAAGGATAAGAATCCC
                              ..|||||||||.||||||||
ATXN10 gRNA                   AUACAAAGGAUCAGAAUCCC
```

Figure S4. Coverage plots for HD samples. Genome wide coverage plots for the 11 HD samples.

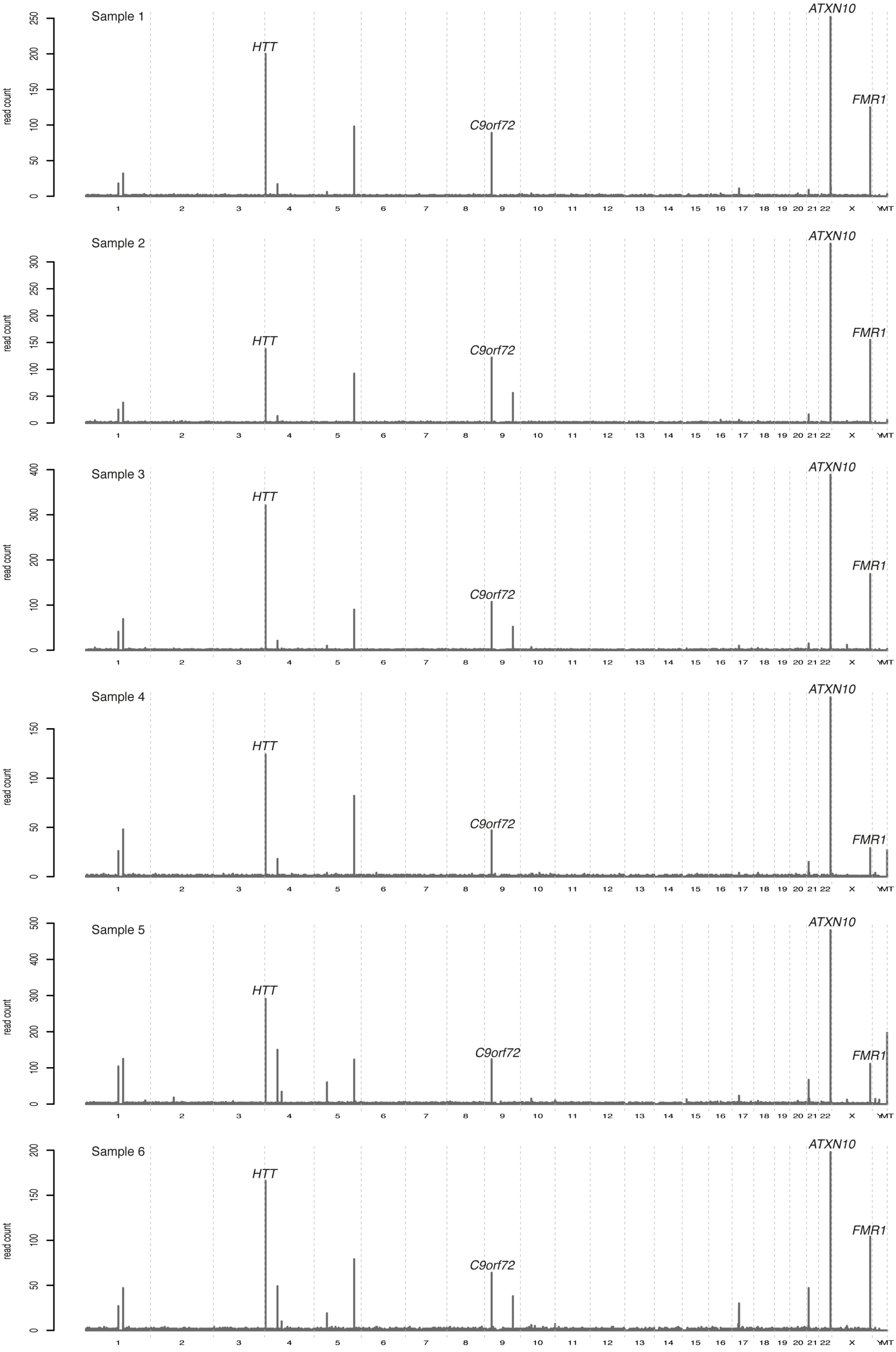

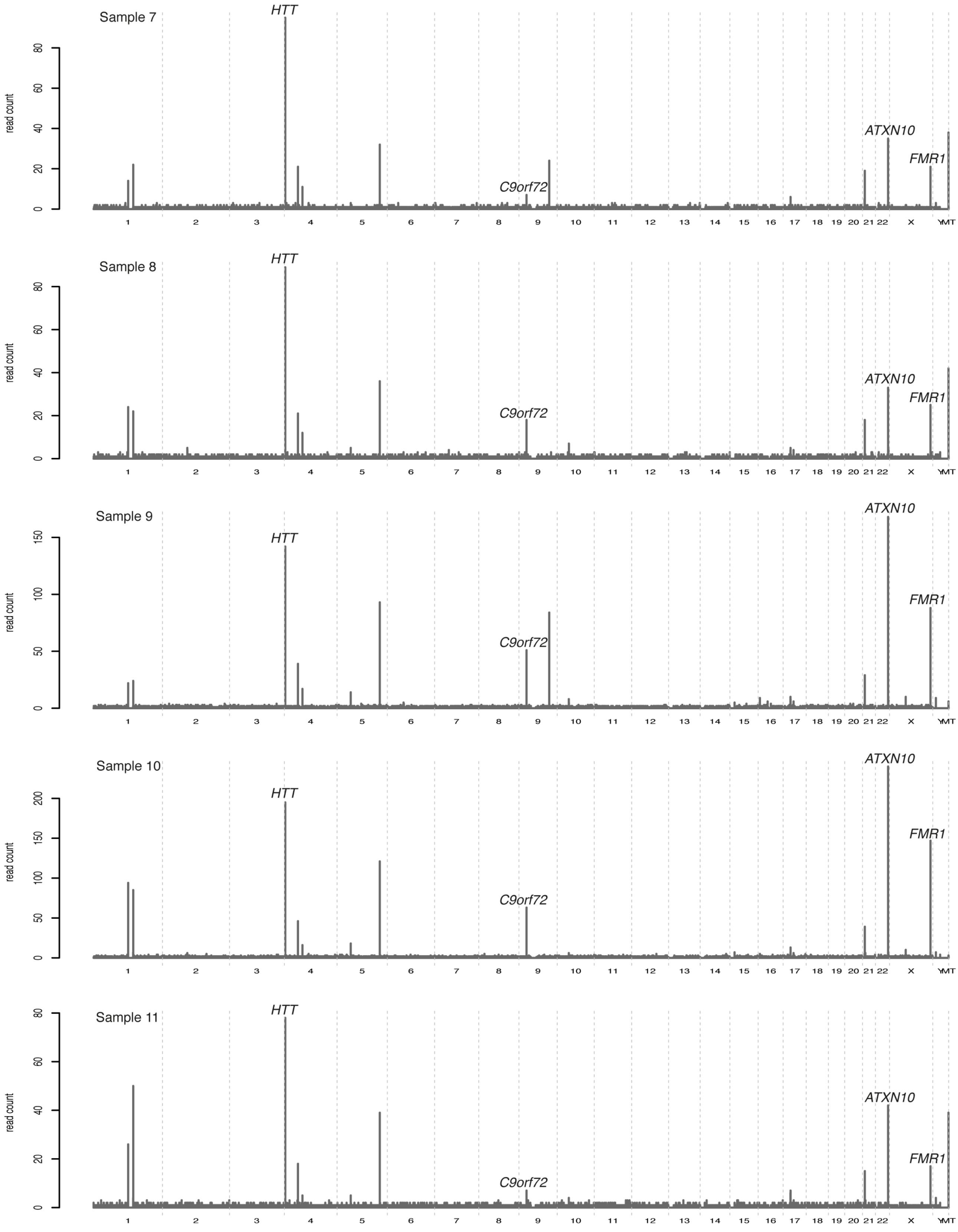
Sample 7
HTT
C9orf72
ATXN10
FMR1
read count
Sample 8
HTT
C9orf72
ATXN10
FMR1
read count
Sample 9
HTT
C9orf72
ATXN10
FMR1
read count
Sample 10
HTT
C9orf72
ATXN10
FMR1
read count
Sample 11
HTT
C9orf72
ATXN10
FMR1
read count
1 2 3 4 5 6 7 8 9 10 11 12 13 14 15 16 17 18 19 20 21 22 X YMT

Figure S5. Allelic distribution of CAG repeats and repeat-content plots for 11 HD samples. The histograms in the left column show the allelic CAG distribution for all HD samples. The repeat-content plots in the right column show CAG repeats in red and CCG repeats in blue. Sequences that are neither CAG nor CCG are shown in grey.

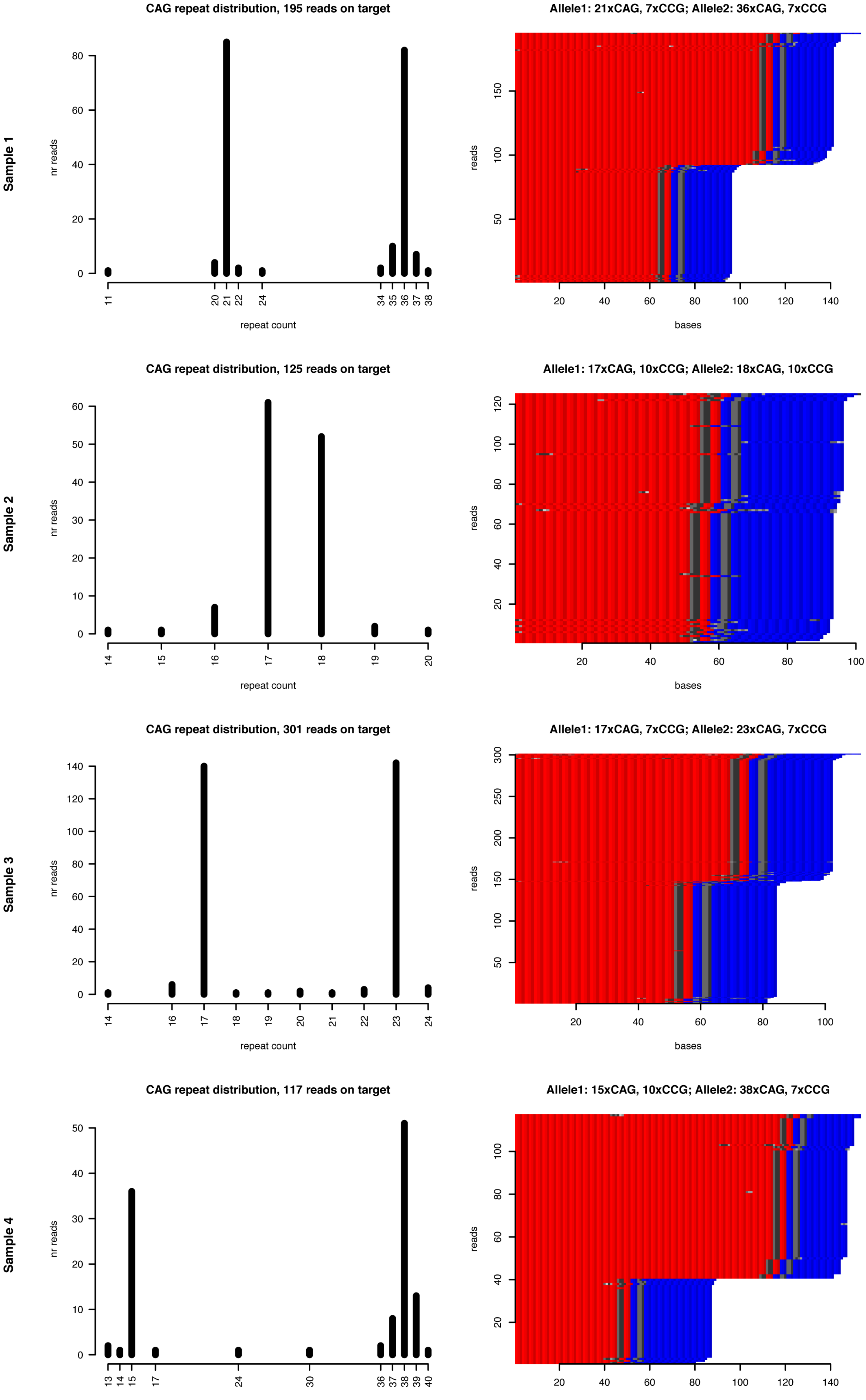

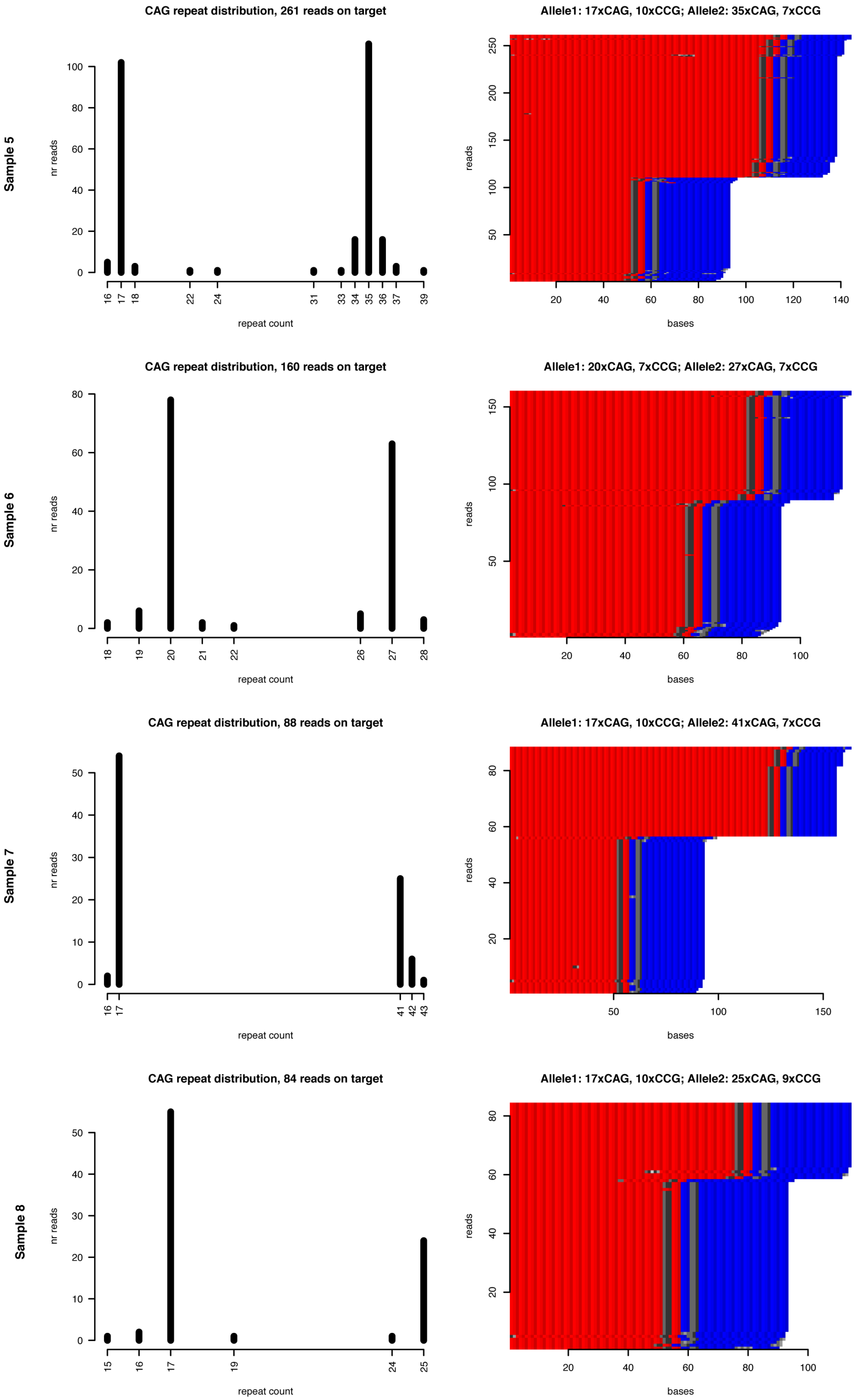
Sample 5
CAG repeat distribution, 261 reads on target
nr reads
repeat count
Allele1: 17xCAG, 10xCCG; Allele2: 35xCAG, 7xCCG
reads
bases
Sample 6
CAG repeat distribution, 160 reads on target
nr reads
repeat count
Allele1: 20xCAG, 7xCCG; Allele2: 27xCAG, 7xCCG
reads
bases
Sample 7
CAG repeat distribution, 88 reads on target
nr reads
repeat count
Allele1: 17xCAG, 10xCCG; Allele2: 41xCAG, 7xCCG
reads
bases
Sample 8
CAG repeat distribution, 84 reads on target
nr reads
repeat count
Allele1: 17xCAG, 10xCCG; Allele2: 25xCAG, 9xCCG
reads
bases

Sample 9

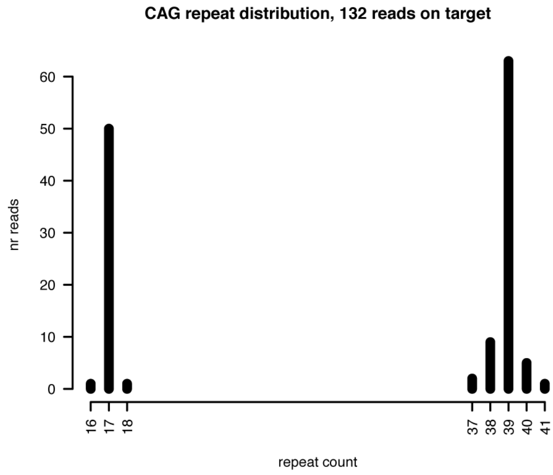

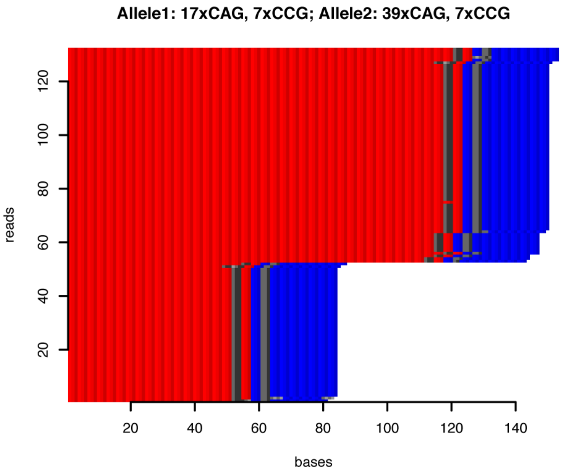

Sample 10

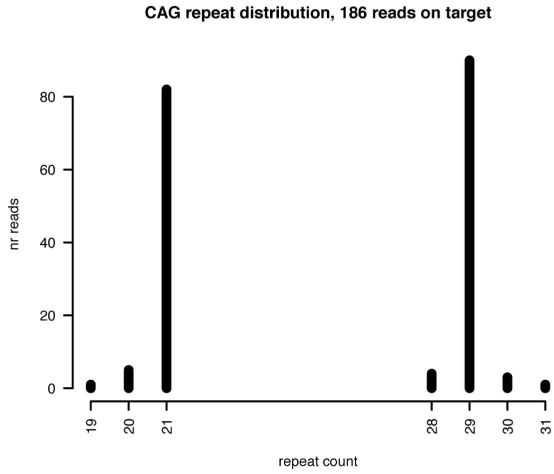

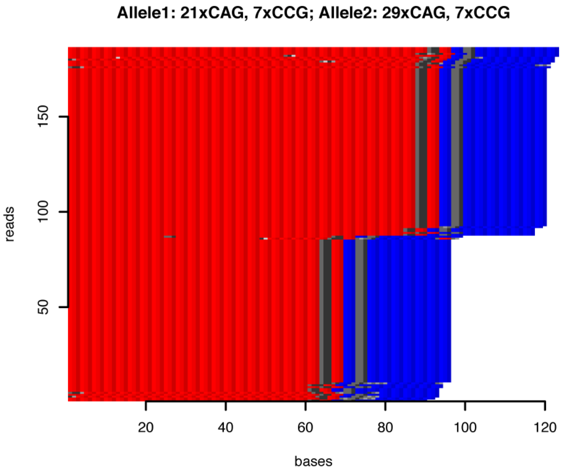

Sample 11

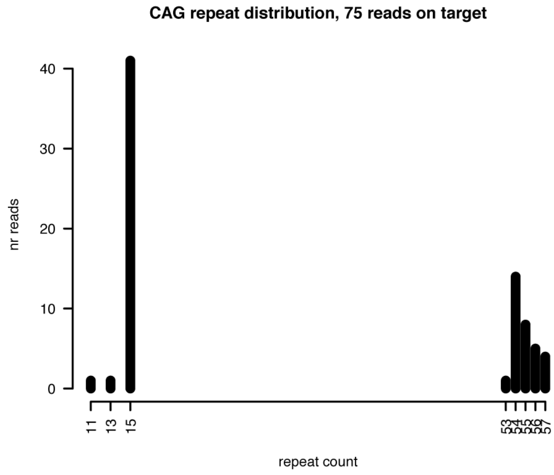

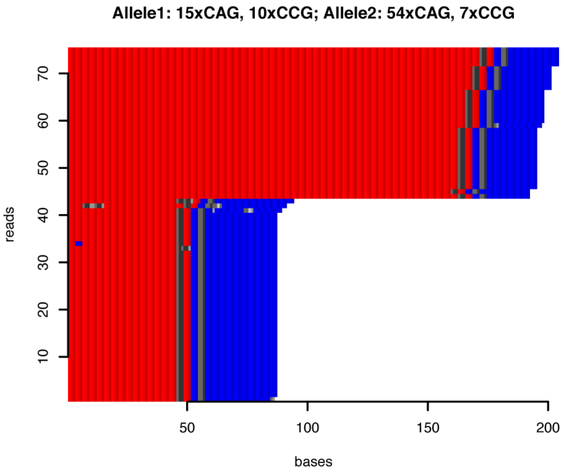

Figure S6. Fragment analysis results for sample 11. Fragment analysis result for sample 11 showing two alleles with 15 and 54 CAG repeats respectively.

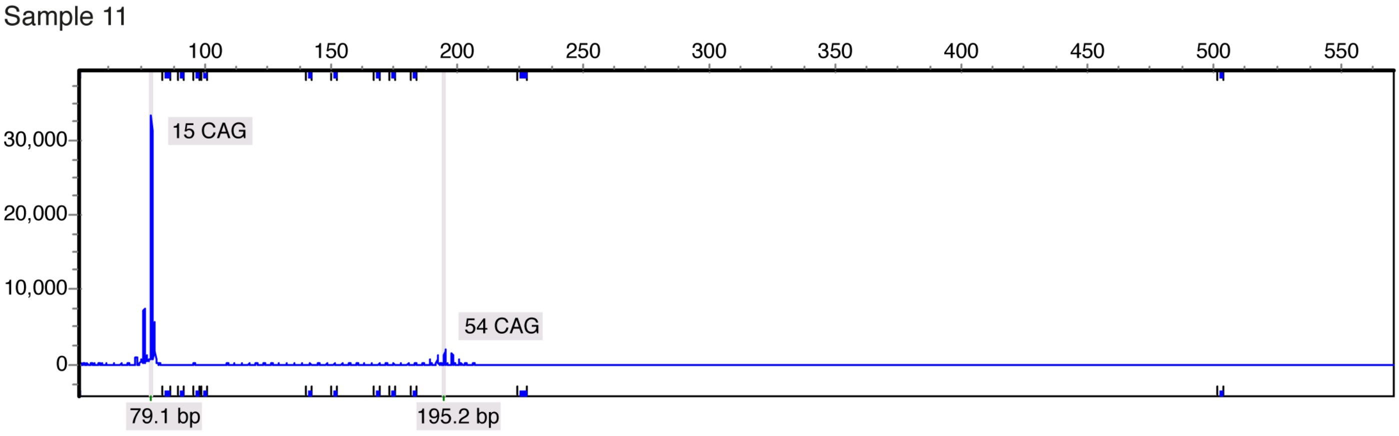

Supporting Tables

Table S1. Sample library preparation information.

Sample	Input DNA (μg)	Amount into capture (ng)	Complexity reduction restriction enzymes	gRNAs
Replicate 1	20	764	KpnI, EcoRV, MfeI, SpeI	*ATXN10*, *FMR1*, *HTT*, *C9orf72a*
Replicate 2	20	743	KpnI, EcoRV, MfeI, SpeI	*ATXN10*, *FMR1*, *HTT*, *C9orf72a*
Replicate 3	20	398	KpnI, EcoRV, MfeI, SpeI	*ATXN10*, *FMR1*, *HTT*, *C9orf72a*
Replicate 4	20	588	KpnI, EcoRV, MfeI, SpeI	*ATXN10*, *FMR1*, *HTT*, *C9orf72a*
Replicate 5	20	668	KpnI, EcoRV, MfeI, SpeI	*ATXN10*, *FMR1*, *HTT*, *C9orf72a*
Replicate 6	20	651	KpnI, EcoRV, MfeI, SpeI	*ATXN10*, *FMR1*, *HTT*, *C9orf72a*
Sample 1	5.5	570	KpnI, EcoRV	*ATXN10*, *FMR1*, *HTT*, *C9orf72b*
Sample 2	4.9	468	KpnI, EcoRV	*ATXN10*, *FMR1*, *HTT*, *C9orf72b*
Sample 3	5.1	378	KpnI, EcoRV	*ATXN10*, *FMR1*, *HTT*, *C9orf72b*
Sample 4	3.8	258	KpnI, EcoRV	*ATXN10*, *FMR1*, *HTT*, *C9orf72b*
Sample 5	5.7	792	KpnI, EcoRV	*ATXN10*, *FMR1*, *HTT*, *C9orf72b*
Sample 6	5.7	588	KpnI, EcoRV	*ATXN10*, *FMR1*, *HTT*, *C9orf72b*
Sample 7	5.9	714	KpnI, EcoRV	*ATXN10*, *FMR1*, *HTT*, *C9orf72b*
Sample 8	7.7	762	KpnI, EcoRV	*ATXN10*, *FMR1*, *HTT*, *C9orf72b*
Sample 9	5.7	582	KpnI, EcoRV	*ATXN10*, *FMR1*, *HTT*, *C9orf72b*
Sample 10	5.9	666	KpnI, EcoRV	*ATXN10*, *FMR1*, *HTT*, *C9orf72b*
Sample 11	7.4	786	KpnI, EcoRV	*ATXN10*, *FMR1*, *HTT*, *C9orf72b*

Table S2. Reads on-target and total CCS reads for HEK 293 replicates.

			C9orf72 (GGGGCC = 3)*			*ATXN10* (ATTCT = 14)*			*FMR1* (CGG = 19)*			*HTT* (CAG = 19)*		
**Sample**	**CCS reads**	**Total CCS reads on-target**	**CCS reads on-target**	**$(GGGGCC)_n$ Allele 1**	**$(GGGGCC)_n$ Allele 2**	**CCS reads on-target**	**$(ATTCT)_n$ Allele 1**	**$(ATTCT)_n$ Allele 2**	**CCS reads on-target**	**$(CGG)_n$ Allele 1**	**$(CGG)_n$ Allele 2**	**CCS reads on-target**	**$(CAG)_n$ Allele 1**	**$(CAG)_n$ Allele 2**
Replicate 1	31218	1401	59	2	4	580	16	16	480	28	29	282	17	17
Replicate 2	27408	1906	114	2	4	1143	16	16	400	28	29	249	17	17
Replicate 3	11194	324	26	2	4	147	16	16	90	28	29	61	17	17
Replicate 4	38879	1863	98	2	4	1079	16	16	426	28	29	260	17	17
Replicate 5	39863	1559	80	2	4	851	16	16	426	28	29	202	17	17
Replicate 6	29731	1386	76	2	4	727	16	16	383	28	29	200	17	17

* Number of repeats in human reference genome GRCh38

Table S3. Reads on-target and total CCS reads for 11 patient samples.

			C9orf72 (GGGGCC = 3)*			*ATXN10* (ATTCT = 14)*			*FMR1* (CGG = 19)*			*HTT* (CAG = 19)*		
**Sample**	**CCS reads**	**Total CCS reads on-target**	**CCS reads on-target**	**$(GGGGCC)_n$ Allele 1**	**$(GGGGCC)_n$ Allele 2**	**CCS reads on-target**	**$(ATTCT)_n$ Allele 1**	**$(ATTCT)_n$ Allele 2**	**CCS reads on-target**	**$(CGG)_n$ Allele 1**	**$(CGG)_n$ Allele 2**	**CCS reads on-target**	**$(CAG)_n$ Allele 1**	**$(CAG)_n$ Allele 2**
Sample 1	5902	595	85	2	2	214	14	15	101	28	30	195	21	36
Sample 2	7317	644	114	2	2	295	13	14	110	27	28	125	17	18
Sample 3	9892	868	96	2	2	337	12	15	134	28	29	301	17	23
Sample 4	5775	333	43	7	15	155	14	17	18	34	35	117	15	38
Sample 5	33415	827	109	2	2	396	13	14	61	28	28	261	17	35
Sample 6	11590	452	60	2	8	164	14	17	68	22	28	160	20	27
Sample 7	4862	131	7	2	5	27	14	14	9	23	27	88	17	41
Sample 8	6025	137	16	2	2	25	14	14	12	35	37	84	17	25
Sample 9	10624	378	49	2	2	143	13	14	54	28	29	132	17	39
Sample 10	13500	548	60	2	2	202	14	16	100	28	35	186	21	29
Sample 11	5852	120	6	2	4	28	13	14	11	29	29	75	15	54

* Number of repeats in human reference genome GRCh38
